# Antimicrobial Secondary Metabolites from the Mangrove Plants of Asia and the Pacific

**DOI:** 10.3390/md20100643

**Published:** 2022-10-15

**Authors:** Mazdida Sulaiman, Veeranoot Nissapatorn, Mohammed Rahmatullah, Alok K. Paul, Mogana Rajagopal, Nor Azizun Rusdi, Jaya Seelan Sathya Seelan, Monica Suleiman, Zainul Amiruddin Zakaria, Christophe Wiart

**Affiliations:** 1 Department of Chemistry, Faculty of Science, University of Malaya, Kuala Lumpur 50603, Malaysia; 2 School of Allied Health Sciences and World Union for Herbal Drug Discovery (WUHeDD), Walailak University, Nakhon Si Thammarat 80160, Thailand; 3 Department of Biotechnology & Genetic Engineering, University of Development Alternative, Dhaka 1207, Bangladesh; 4 School of Pharmacy and Pharmacology, University of Tasmania, Hobart, TAS 7001, Australia; 5 Faculty of Pharmaceutical Sciences, UCSI University, Kuala Lumpur 56000, Malaysia; 6 Institute for Tropical Biology & Conservation, University Malaysia Sabah, Kota Kinabalu 88400, Malaysia; 7 Department of Biomedical Sciences, Faculty of Medicine and Health Sciences, University Malaysia Sabah, Kota Kinabalu 88400, Malaysia

**Keywords:** mangrove plants, shrimp farming, natural products, antibacterial, antifungal, antiviral, Asia, Pacific

## Abstract

Microbes such as the White Spot Syndrome Virus account for severe losses in the shrimp farming industry globally. This review examines the literature on the mangrove plants of Asia and the Pacific with antibacterial, antifungal, or antiviral activities. All of the available data published on this subject were collected from Google Scholar, PubMed, Science Direct, Web of Science, ChemSpider, PubChem, and a library search from 1968 to 2022. Out of about 286 plant species, 119 exhibited antimicrobial effects, and a total of 114 antimicrobial natural products have been identified including 12 with MIC values below 1 µg/mL. Most of these plants are medicinal. The mangrove plants of Asia and the Pacific yield secondary metabolites with the potential to mitigate infectious diseases in shrimp aquaculture.

## 1. Introduction

The global shrimp and prawn aquaculture industry is regularly threatened by outbreaks of microbial infections [1] that require antibiotics, antifungals, and antiviral agents participating in the selection of multidrug-resistant strains of microbes, pausing the grim scenario of the emergence of a “superbug” that could wipe out the global supply of penaeids [2]. In this context, there is an urgent necessity to search for antimicrobial agents with original chemical frameworks, and such molecules could come from the flora of Asia and the Pacific, which is the oldest, largest, and richest on Earth, especially seashores, tidal rivers, and mangrove plants.

Mangroves are ecosystems of the tropical and subtropical seashores, estuaries, and tidal rivers characterized by a halophytic flora of mainly trees and shrubs divided into true mangrove or mangrove-associated species. True mangrove species are restricted to mangroves whereas mangrove-associated species are found along the seashores, and even inland. There are estimates of about 54 true mangrove plant species and 60 mangrove-associated species globally, which are home to shrimps, prawns, crabs, and fish [3]. Most mangrove species grow in Asia and the Pacific [4]. Examples of true mangrove plant species are *Excoecaria agallocha* L. (land zone), *Bruguiera gymnorhiza* (L.) Savigny, *Rhizophora stylosa* Griff. (intermediate zone), *Avicennia alba* Bl, and *Aegiceras corniculatum* (L.) Blanco (fringing zone) [5]. Even though most of the global fish catches are directly or indirectly dependent on mangroves, these are on their way to extinction due to logging, agriculture, aquaculture, and urbanization, with an estimate of about 2–8% loss of surface per year [6]. Shrimps, prawns, and fish farming are the greatest threat to mangroves with, for example, approximately half of the 279,000 ha of mangroves in the Philippines lost from 1951 to 1988 [7]. Another aggravating factor is global warming, and consequently, a rise in sea levels that interfere with the growth of true mangrove plants. 

Most plants in mangroves are Angiosperms organized phylogenetically into 11 major taxa or clades organized in three groups: (i) Basal Angiosperms: Protomagnoliids, Magnoliids, Monocots, Eudicots; (ii) Core Angiosperms: Core Eudicots, Rosids, Fabids, Malvids; and (iii) Upper Angiosperms: Asterids, Lamiids, and Campanulids. Within each clade, plants yield specific secondary metabolites to control and even communicate with phytopathogenic bacteria and fungi. Plants are challenged by phytopathogenic bacteria, fungi, and viruses and produce a vast array of antimicrobial secondary metabolites [8]. These antimicrobial principles fall into two main categories: phytoanticipins and phytoalexins. Phytoanticipins are antimicrobials present in plant tissues before pathogen challenges or inactive immediate precursors of phytoalexins [8]. 

Phytoanticipins and phytoalexins are mainly either phenolics, terpenes, or alkaloids with various levels of solubility in water and are extractable with water, polar organic (methanol, ethanol), mid-polar solvents (chloroform, dichloromethane, ethyl acetate), and non-polar solvents (hexane, petroleum ether) [9]. The measurement of the antibacterial and antifungal strength of extracts and secondary metabolites in vitro is quantitatively based on the minimum inhibiting concentration (MIC) and several thresholds of activity have been proposed [10]. Qualitatively, antibacterial and antifungal strength are appreciated by halos developed around a paper disc or an agar well expressed in the inhibition zone diameter (IZD) [10].

Colette et al. (2022) noted that the presence of *Atriplex jubata* S. Moore evoked some levels of remediation in the shrimp farms of New Caledonia [11] and this review aims to attempt to answer the following points: What is the current knowledge on the distribution of antibacterial, antifungal, and antiviral principles from the mangrove plants of Asia and the Pacific? What are the strongest antimicrobial principles isolated thus far from these plants? What is the spectrum of activity of the antimicrobial principles? What are the medicinal values of these plants? What is the potential usefulness of these plants as remediation of shrimp farming? We hypothesize that a shrimp or prawn farming system preserving healthy mangroves could be a mean to solve the increasing problem of infection.

## 2. Distribution of Antibacterial, Antifungal, and Antiviral Principles Various Mangrove Plants

The enumeration of mangrove and mangrove-associated plants is provided in Table S1, and the chemical structures of the antimicrobial secondary metabolites identified from these plants is given Figure S1.

### 2.1. Subclass Lycopodidae

The only lycopod associated with mangroves is *Lycopodium carinatum* Desv. ex Poir., for which no antimicrobial activities have been recorded thus far.

### 2.2. Subclass Polypodiidae 

Aqueous and polar organic extracts of ferns of the mangrove are moderately broad-*spectrum* antibacterial and antifungal (Table 1). Data on the antiviral properties of ferns are lacking. The methanol extract of *Stenochlaena palustris* (Burm. f.) Bedd. (25 µL/6 mm disc of a 100 mg/mL solution) evoked halos against *Staphylococcus aureus*, *Bacillus subtilis*, *Escherichia coli*, *Klebsiella pneumoniae*, *Salmonella typhi*, *Penicillium chrysogenum*, *Aspergillus niger*, and *Saccharomyces cerevisae* [12]. From the leaves of this fern was identified the flavonol glycoside stenopalustroside A (**1**), which strongly repressed the *Staphylococcus epidermidis* [13]. Antimicrobials in this subclass are mainly phenolics. Other ferns with broad-spectrum antibacterial and antifungal properties are *Nephrolepis biserrate* (Sw.) Schott, *Drynaria* *quercifolia* (L.) J. Sm., *Drymoglossum piloselloides* (L.) Presl., *Pyrrosia piloselloides* (L.) Farw., *Microsorum punctatum* (L.) Copel. [14,15,16], *Phymatosorus scolopendria* (Burm. f.) Pic. Serm. [17], *Platycerium coronarium* (O.F. Müll.) Desv. [18], and the true mangrove fern *Acrostichum aureum* L. [19,20,21]. Of note, the ethyl acetate extract of roots of *Acrostichum speciosum* L., which is a true mangrove fern, was bactericidal for *E. coli* with the MIC/MBC of 40/40 µg/mL [21].

### 2.3. Subclass Cycadaceae 

The ethyl acetate extract of *Cycas rumphii* Miq. developed halos against *Staphylococcus albus* whereas the methanol extract (20 mg/mL solution per disc) hampered the growth of *S. aureus* and *E. coli* [22]. Later, the methanol extract of leaves (paper disc impregnated with 20 mg/mL solution) repressed *S. aureus* (ATCC 25953) [22]. Note that the Cycadaceae have not been much studied for their antimicrobial effects (Table 1) [23].

### 2.4. Subclass Magnoliidae

Mangrove plants in this subclass produce most of, and a broad spectrum of antimicrobial secondary metabolites.

#### 2.4.1. Clade Protomagnoliids

Plants in the clade are not found in mangroves.

#### 2.4.2. Clade Magnoliids

Plants in this clade are not common in mangroves and principally yield antimicrobial isoquinoline alkaloids and lignans (Table S2). In the Lauraceae, the filamentous climber *Cassytha filiformis L. yields* the aporphine dicentrine (**2**), which inhibits the growth of *Cladosporium clodosporioides* (Table S2) [24]. *Hernandia nymphaeifolia* (C. presl.) Kubitzki (Hernandiaceae) produces the dibenzyl butyrolactone lignan deoxypodophyllotoxin (**3**), which is strongly active against HSV (Table S2) [25]. In the family Annonaceae, the hexane extract of stem bark of *Annona glabra* L. exhibited antibacterial and antifungal properties on account of kaurane diterpenes (Table S2) [26].

#### 2.4.3. Clade Monocots

Plants in this clade are mainly mangrove-associated with organic extracts being moderately broad-spectrum antibacterial and antifungal and producing mainly antimicrobial phenolics (Table 2). For instance, the ethanol extract of rhizomes of *Lasia spinosa* (L.) Thwaites developed halos with *S. aureus*, *S. epidermidis*, *S. pyogenes*, *S. dysenteriae*, *E. coli*, *V. cholerae*, *E. aerogenes*, *P. aeruginosa*, *C. albicans*, *A. niger*, and *S. cerevisae* (500 µg/disc) [27,28]. Other instances are *Phoenix paludosa* Roxb. [29,30], *Saribus rotundifolius* (Lam.) Bl. [31], *Cyperus scariosus* R. Br. [32], *Eleocharis dulcis* (Burm. f.) Trin. ex Hensch. [33,34], *Pandanus tectorius* Parkinson [35], the true mangrove *Nypa fruticans* Wurm. [35], *Areca catechu* L. [31], *Phragmites vallatoria* Veldkamp [36], *Ruppia maritima* L. [37], and *Flagellaria indica* L. [38]. The ethanol extract of *Flagellaria indica* L. at the concentration of 12.5 µg/mL repressed DV by 45.5% [39,40]. In the family Orchidaceae, an aqueous extract of *Aerides odoratum* Reinw. ex Bl. repressed the *E. coli* [41] and the chloroform extract of pseudobulbs of *Cymbidium finlaysonianum* Wall. ex Lindl. moderately retrained *T. Mentagrophytes* (MIC: 250 µg/mL) [42]. From this orchid, the phytoalexin stilbene batatasin III (**4**) was active against Gram-positive bacteria [43] as well as phytopathogenic filamentous fungi [44]. Gigantol (**5**) and batatasin III exhibited meek activity with HSV-1 and -2 [45]. The phenanthrene moscatin (**6**) from *Dendrobium moschatum* (Buch. -Ham.) Sw. is a moderate antibacterial [46]. Other examples of antibacterial and antifungal phenolics from the Monocots are meridinol (**7**) [47], tricin (**8**) [48,49], and naringenin (**9**) [50,51] (Table 2).

#### 2.4.4. Clade Core Eudicots

Plants in this clade are not found in mangroves.

#### 2.4.5. Clade Core Eudicots

Plants in this clade are not found in mangroves.

#### 2.4.6. Clade Rosids

The ethanol extract of the leaves of *Cayratia trifolia* (L.) Domin (*Vitaceae)* inhibited the growth of *S. aureus* [52]. This climber produces antibacterial and antifungal as well as antivirals as in ɛ -viniferin (**12**) piceid (**13**), and resveratrol (**14**) (Table S2), [53,54,55,56,57,58,59].

#### 2.4.7. Clade Fabids

Fabids principally yield antimicrobial phenolics (Table S2).

*Order Malpighiales:* Organic polar, mid-polar, and non-polar extracts of *Calophyllum inophyllum* L. (Clusiaceae) are broadly antimicrobial [60,61,62]. Of note, the methanol extract of latex very strongly hindered *S. aureus* with an IC_50_ of 1.1 µg/mL and *Trichophyton rubrum* with an IC_50_ of 3.3 µg/mL [63]. The hexane extract of seeds strongly restrained HIV-1 at the concentration of 10 µg/mL [64]. Inophyllum B (**15**), inophyllum B acetate (**16**), and inophyllum P (**17**) from the leaves blocked HIV reverse transcriptase respectively, while inophyllum B (**15**) and P (**17**) inhibited HIV with IC_50_ values of 1.4 and 1.6 µM, respectively (Table S2) [61].

In the Euphorbiaceae, extracts of leaves of *Excoecaria agallocha* L. moderately repressed a broad array of bacteria and yeasts [64,65,66,67]. The ethanol extract of leaves inhibited the replication of the ECMV (EC_50_:16.7 µg/mL), HIV (EC_50_: 7.3 µg/mL), NDV, and SFV [68]. This vesicant tree yields 12-deoxyphorbol 13-(3*E*,5*E*-decadienoate) (**18**) with very strong antiretroviral effects (Table S2) [69]. *Suregada glomerulata* (Bl.) Baill. yields the alkaloid 5*β*-carboxymethyl-3*α*-hydroxy-2*β*-hydroxymethyl-1- methylpyrrolidine (**19**), which curbed HIV-1 replication (Table S2) [70].

Plants in the Rhizophoraceae are tanniferous and have antibacterial activities as in *Bruguiera cylindrica* (L.) Bl. [71], *Bruguiera gymnorhiza* (L.) Savigny [72], *Bruguiera sexangula* (Lour.) Poir., *Ceriops decandra* Griff.) Ding Hou [28,63], *Ceriops tagal* (Perr.) C.B. Rob. [71], *Kandelia candel* (L.) Druce [73,74], *Rhizophora apiculata* Bl. [75], and *Rhizophora stylosa* Griff. [71,76]. The hydrolysable tannin fraction of the bark of the latter weakly inhibited the growth of *A. calcoaceticus*, *B. lichenifornis*, *P. mirabilis*, and *S. saprophyticus* [77]. Other antibacterials in this family are 2,6-dimethoxy-*p*-benzoquinone (**20**) as well as gallic acid (**21**) [78,79] (Table S2), [78,79,80,81,82,83,84,85,86,87,88,89,90,91,92,93].

16-Hydroxypimar-8(14)-en-15-one (**22**) from the roots of *Ceriops tagal* (Perr.) C.B. Rob. Moderately restrained a broad-spectrum of bacteria (Table S2) [80]. Diterpenes are often liposoluble, explaining perhaps the suppression of a broad-spectrum of bacteria and fungi including *B. pumilus* with a MIC value of 15.6 µg/mL by the benzene extract of the wood of *C. decandra* [94]. The presence of tannins and phenolics most probably account for the antiviral effects observed in *B. cylindrica*, *Rhizophora mucronata* Lam., *R. apiculata*, *B. gymnorhiza* [72], *C. decandra* [68]. Other examples of water soluble antibacterials are the cyclohexylideneacetonitrile derivatives from *B. gymnorhiza*, which strongly repressed HBV [69].

*Order Fabales:* Aqueous, polar and mid-polar extracts of Fabaceae are moderately broad-spectrum antibacterial and antifungal, as observed with *Caesalpinia bonduc* (L.) Roxb [95] (Table S2) [96]. The methanol extract of the seed coat of this climber strongly restrained *P. aeruginosa, S. aureus, and B. cereus (MIC: 22 µg/mL)* [97]. *This extract given to Wistar rats* subcutaneously at a dose of 25 mg/kg body weight once a day for 10 days evoked a reduction in lung abscesses induced by *P. aeruginosa* [97]. The active principle here are diterpenes including bondenolide (**23**) [96] and neocaesalpin P **(24**) [98].Other examples of Fabaceae yielding antibacterial or antifungal organic polar or mid-polar extracts are- *Canavalia maritima* Thouars [99], the true mangrove tree *Cynometra iripa* Kostel. [100], *Cynometra* ramiflora Miq. [101], *Derris scandens* (Aubl.) Pittier [17], *Derris trifoliata* Lour. [102], *Inocarpus fagifer* (Parkinson) Fosb. [17], *Sindora siamensis* Teysm. ex Miq. [103], *Pongamia pinnata* (L.) Pierre [104], and *Cathormion umbellatum* (Vahl) Kosterm. [105]. *Plants in this family yield antibacterial and/or antifungal* isoflavonoids such as lupalbigenin (**25**) and derrisisoflavone A (**26**) from *Derris scandens* (Aubl.) Pittier [106,107,108,109] (Table S2) [107,109,110]. Other examples are santal (**27**), scandenin A (**28**) dalpanitin (**29**), vicenin 3 (**30**), derrisisoflavone C (**31**), and 5,7,4′-trihydroxy-6,8-diprenylisoflavone (**32**) [107]. Organic and aqueous extracts in this family are often antiviral, as in *D. scandens* with HSV-1 (IC_50_: 60 μg/mL) PV and MV as well as *Cynometra ramiflora* Miq. with DV-2 [111], and *Derris trifoliata* Lour. with HIV [112,113]. As for antiviral principles, isoflavone deguelin (**33**) was active against HCMV [113,114] whereas rotenone (**34**) restrained HSV-1 and -2. *D. trifoliata* yields the strong antibacterial and anticandidal lupinifolin (**35**) (Table S2) [115,116,117]

*Order Fagales****:*** Organic polar and mid-polar extract of fruits and leaves of the tanniferous *Casuarina equisetifolia* L. (Casuarinaceae) are broadly antibacterial and antifungal [118,119,120]. 

*Order Rosales:* In the Moraceae, the methanol extract of the bark of *Ficus microcarpa* L.f. (40 µL of a 10 mg/mL solution on 6 mm disc) developed halos with *B. brevis*, *B. cereus*, *B. subtilis*, *E. coli*, and *A. polymorph* [121]. From this tree, the flavanols (+) (2*R*,3*S*) afzelechin (**36**) and (-)(2*R*,3*R*) epiafzelechin (**37**) weakly repressed HSV-1 (Table S2) [122].

#### 2.4.8. Clade Malvids

Antimicrobials in this vast Clade are diverse (Table S2).

*Order Myrtales:* Polar and mid-polar organic extracts of *Combretum quadrangulare* Kurz (Combretaceae) [123] and *Terminalia catappa* L. (Combretaceae) are antibacterial and anticandidal [124,125] probably due to ellagitannins such as corilagin (**38**) [81,126] (Table S2) [127,128] and other phenolics. Phenolic fraction from the fruits of *T. catappa* strongly repressed *S. aureus*, *B. subtilis*, *E. faecalis*, and *L. monocytogenes* with the MIC values of 15.6, 15.6, 7.8, and 15.6 µg/mL, respectively [129]. Other antimicrobials in this family are triterpenes, probably explaining the strong activity of the hexane extract of *Lumnitzera racemosa* Willd. with *B. cereus* and *E. coli* [21]. The ethanol extract of barks of this shrub repressed NVD, VV, EMCV, and SFV [130]. Aqueous extracts from Combretaceae plants are often antiviral, as in the pericarps of *T. catappa* with HSV-2 [131] or *C. quadrangulare* blocking HIV integrase with the IC_50_ of 2.9 µg/mL [132]. 

The methanol extract of the true mangrove shrub *Pemphis acidula* J.R. & G. Forst (Lythraceae) hindered a broad-spectrum of bacteria [133,134]. Essential oils of *Melaleuca cajuputi* Roxb. and *Melaleuca quinquenervia* (Cav.) S.T. Blake (*Myrtaceae*) are strongly and broadly antibacterial and antifungal [135,136,137,138]. The essential oil of *M. quinquenervia* repressed *Phytophthora cactorum* [139] and was strongly fungicidal for filamentous fungi [140].

In the family Sonneratiaceae, polar and mid-polar organic and aqueous extracts of the true mangrove trees *Sonneratia apetala* Buch-Ham., *Sonneratia griffithii* Kurtz., and *Sonneratia ovata* Back. are bacterial and antifungal [21,141,142,143,144,145]. *S. griffithii* yields strongly antibacterial lupane triterpenes such as 3β-hydroxy-lup-9(11),12-diene, 28-oic acid (**39**), lupeol (**40**), and lupan-3β-ol (**41**) (Table S2) [146]. Antiviral triterpenes are present in Sonneratiaceae plants [147].

*Order Brassicales:* Polar organic extracts of *Azima sarmentosa* (Bl.) B. & H and *Azima tetracantha* Lam. (Salvadoraceae) inhibited the growth of bacteria and fungi [148,149].

*Order Malvales:* In the family Malvaceae, organic polar extracts of *Hibiscus tiliaceus* L. and *Thespesia populnea* (L.) Soland. ex Correa restrained a broad array of bacteria [150,151]. *T. populnea* yields the cadalane sesquiterpenes populene C (**42**) and D (**43**), mansonone D (**44**) and E (**45**), 7-hydroxycadalene (**46**), gossypol (**47**), and (+) 6,6’-methoxygossypol (**48**) with strong activity toward Gram-positive bacteria (Table S2) [152]. The ethanol extract of flowers of *T. populnea* strongly hindered VSV, CV B4, and RSV (EC_50_: 20 μg/mL) [153] whereas the methanol extract of *Malachra capitata* *(L.) L*. was active against the FMDV [154]. The petroleum ether extract of the leaves of *Kleinhovia hospita* L. strongly retrained *E. coli* and *A. jejunii* with the MIC values of 35.7 and 38 µg/mL, respectively [155,156], while the ethanol extract of the bark yielded a MIC value of 4 µg/mL with *S. aureus* [17]. From this plant, the steroids (9*R*,10*R*, 23*R*)-21,23:23,27-diepoxycycloarta-1,24-diene-3,27-dione (**49**) and (9*R*,10*R*,21*S*,23*R*)-21/23,23/27-diepoxy-21-methoxycycloartan-1,24-diene-3,27-dione (**50**) are strongly active (Table S2) [156]. 

Sterculiaceae plants are often antimicrobial as in the dichloromethane extract *Heritiera littoralis* Aiton with *M. madagascariense* and *M. indicus* [157,158]. From this tree, the flavonol glycoside afzelin (**51**) is strongly antibacterial and antiviral (Table S2) [55,159,160,161,162,163].

Other antimicrobial principles in this true mangrove tree are taraxerol (**52**), friedelin (**53**), and astilbin (**54**) [164]. The ethanol extract of the bark of *Heritiera fomes* Buch. Ham. developed halos with *S. epidermidis*, *S. pyogenes*, *E. coli*, *E. aerogenes*, *Pseudomonas sp*. [28], and *K. rhizophilia* [164]. 

*Order Sapindales:* Organic polar extracts of the true mangrove trees *Aglaia cucullata* Pellegr., *Xylocarpus granatum* J. Koenig, and *Xylocarpus moluccensis* (Lam.) M. Roem (Meliaceae) displayed antibacterial properties [28,165,166] (Table S2) [144–146,148). Phytoalexins in this family are limonoids such as in the antiretroviral sundarbanxylogranin B (**55**) from the seeds of *X. granatum* [167] or thaixylomolin I (**56**) and K (**57**) isolated from the seeds of *X. moluccensis* [168]. Another example is krishnolide A (**58**) [169]. From the latter, moluccensin I from the fruits moderately inhibited the growth of *S. hominis* and *E. faecalis* [170]. The limonoid catabolite dihydrofuranone 3-(1-hydroxyethyl)-2,2-dimethyl-4-butyrolactone (**59**) from the leaves of *X. granatum* is a strong repressor of the phytopathogenic fungi *Blumeria graminis* [171]. 

In the Rutaceae, essential oils of *Acronychia pedunculata* (L.) Miq. and *Limnocitrus littoralis* (Miq.) Swingle are antibacterial and antifungal [172,173,174]. The ethanol extract of the former was active toward *C. albicans*, *A. niger*, and *C. neoformans* [175]. The acridone pharmacophore [176] intercalates into microbial DNA [177] and represses WSSV [178]. *A. pedunculata* yields very strong antistaphylococcal acridone alkaloids [179] as well as the prenylated acetophloroglucinol acrovestone (**60**) [177] (Table S2) [180]. 

Antimicrobials in the family Sapindaceae are mainly triterpene saponins and triterpenes the later soluble lipid. The petroleum ether of *Allophylus cobbe* (L.) Raeusch strongly inhibited the growth of *Shigella sonnei, Salmonella paratyphi*, and *C. neoformans* with the MIC values of 31.2 µg/mL [178,179,180,181]. The methanol extract of the leaves of *Harpullia arborea* (Blanco) Radlk repressed a broad-spectrum of bacteria and fungi [182] whereas the ethanol extract of leaves was active against HCV [183] on probable account of simple phenolic glycosides [184]. 

The methanol extract of leaves of *Quassia indica* (Gaertn.) Nooteboom (Simaroubaceae) developed halos with *E. coli*, *S. aureus*, *A. Niger*, and *C. albicans* [185].

*Order Santalales:* Plants in the Loranthaceae generate antimicrobial phenolics such as the flavonol glycoside quercitrin isolated from the leaves of *Dendrophthoe pentandra* (L.) Miq. (100 µg/mL/6 mm disc) [186]. These are soluble in methanol explaining the antibacterial properties of *Macrosolen cochinchinensis* (Lour.) Tiegh [187] or *Viscum orientale* Willd. [188]. The organic polar extracts of *Olax scandens* Roxb. and *Ximenia americana* L. (Olacaceae) are antibacterial and antifungal [189,190,191]. Phytoalexins here are often polyacetylene fatty acids extractable with non-polar solvent from which the halos developed against *B. subtilis*, *Enterococcus faecalis*, *P. aeruginosa*, and *K. pneumoniae* with the hexane extract of the leaves of *Olax scandens* Roxb. [192]. The methanol extract of the stembark of *X. americana* strongly inhibited the replication of HIV [193].

*Order Caryophyllales:* In the order Caryophyllales, a fatty acid fraction of *Sesuvium portulacastrum* (L.) L. (Aizoaceae) as well as the essential oil moderately hindered a broad-spectrum of bacteria and fungi [194,195]. The ethanol extract of leaves was active against HBV [130]. The polar organic extract of *Salicornia brachiata* Miq. and *Suaeda maritima* (L.) Dumort. (Chenopodiaceae) displayed broad-spectrum antibacterial, antifungal, and antiviral properties [196,197]. The fatty acid fraction of the aerial parts of *S. brachiata* Miq. moderately restrained *B. subtilis S. aureus* and methicillin-resistant *S. aureus* [198]. The ethanol extract of the leaves of *S. maritima* was active against the EMCV [130]. The organic polar extract of the true mangrove tree *Aegialitis rotundifolia* Roxb. and *Limonium tetragonium* Bullock (Plumbaginaceae) are antibacterial and antifungal [199,200]. The methanol extract of the roots of *L. tetragonium* Bullock blocked HIV-1 reverse transcriptase [201].

#### 2.4.9. Clade Asterids

Plants in this clade yield antimicrobial triterpenes (Table S2).

In the order Ericales, the ethanol extract of the bark of the true mangrove tree *Diospyros littorea* (R. Br.) Kosterm. (Ebenaceae) developed halos with *Streptococcus* sp., *S. aureus*, *Aeromonas hydrophila*, and *Vibrio parahaemolyticus* [62]. 

Antimicrobial principles in the Lecythidaceae are mainly triterpene saponins that are soluble in polar organic and aqueous extracts, explaining why plants in the genus *Barringtonia* J.R. Forst. & G. Forst. are antibacterial and antifungal. The methanol extract of leaves of *Barringtonia acutangula* (L.) Gaertn. hindered *C. albicans* and *Candida tropicalis* with the MIC values of 31.2 and 62.5 µg/mL, respectively [202,203]. Other examples include the methanol extract of *Barringtonia asiatica* (L.) Kurz [204] or *Barringtonia racemosa* (L.) Spreng [205,206]. Other antimicrobial principles are oleanane triterpenes such as germanicol caffeoyl ester (**61**), camelliagenone (**62**), and germanicol (**63**) in *B. asiatica* (Table S2) [207] or lupeol (**40**) (Table S2) [208]. These are mainly lipophilic, where activities in non-polar extracts as exemplified with the petroleum ether extract of the stem bark of *B. asiatica* that strongly restrained *B. subtilis* with the MIC value of 25 µg/mL, respectively [209]. Other lipophilic to mid-polar principles in *B. racemosa* are neo-clerodane diterpenes such as nasimalun A (**64**) [204,208,210]. The aqueous extract suppressed HSV-1 (IC_50_: 23 µg/mL) [211].

The ethanol extract of the bark (500 µg/disc) of the true mangrove tree *Aegiceras corniculatum* (L.) Blanco (Aegicerataceae) developed a halo with a broad spectrum of bacteria [28], while the hexane extract of leaves inhibited the growth of *Mycobacterium tuberculosis* (H_37_R_v_) with the MIC value of 50 µg/mL [212]. The oleanane triterpene acornine 2 (**65**) isolated from the bark hindered yeasts and filamentous fungi (Table S2) [212]. The ethanol extract of fruits inhibited NDV and SFV [130].

#### 2.4.10. Clade Lamiids

The Lamiids produce various types of antimicrobials (Table S2).

*Order Boraginales:* The *Cordia dichotoma* G. Forst. (Boraginaceae) methanol extract restrained *S. pyogenes*, *S. aureus*, *E. coli*, *P. aeruginosa*, *A. niger*, and *C. albicans* [213]. *Merrilliodendron megacarpum* Sleumer (Icacinaceae) yields the strongly antifungal pyranoindolizinoquinoline alkaloid camptothecin (**66**) which also restrains a broad spectrum of virus in vitro (Table S2) [214,215,216,217,218,219].

*Order Gentianales:* Plants in the genus *Cerbera* L. (Apocynaceae) are antimicrobial. The ethanol extract of *Cerbera manghas* L. very strongly restrained *E. coli* and *P. aeruginosa* (MIC: 4 µg/mL) [17] and VSV (IC_50_: 0.01 µg/mL) [220]. The methanol extract of seeds of *Cerbera odollam* Gaertn. developed halos with a broad-spectrum of bacteria [221] and the ethanol extract of fruits suppressed *Aspergillus flavus*, *Fusarium oxysporum*, and *Penicillium citrum* [222]. Note that Apocynaceous indole alkaloids are often antistaphylococcal [223]. The ethanol extract of the leaves (500 µg/well) of *Hoya parasitica* (Roxb.) Wall. ex Wight (Asclepiadaceae) inhibited the growth of *S. aureus*, *Proteus sp*., *E. coli*, and *S. sonnei*, and *Shigella dysenteriae* with the IZD of 23, 19, 10, 8, and 20 mm, respectively [224].

In the Rubiaceae, the organic polar and mid-polar extracts of *Guettarda speciosa* L., *Hydnophytum formicarum* Jack, *Morinda citrifolia* L., and *Myrmecodia tuberosa* Jack, and *Guettarda speciosa* L. are antibacterial and antifungal [82,225,226,227,228] (Table S2) [81,162,229,230,231]. Plants in this family yield antimicrobial water soluble iridoid glycosides such as in loganic acid (**67**) from *G. speciosa,* which strongly repressed HCV [228], and asperuloside (**68**) from *M. citrifolia* (Table S2) [77,91,93,220,232,233,234]. Other antimicrobial principles are caffeic acid derivatives including the antiviral 4,5-di-*O*-caffeoylquinic acid (**69**) [229,235] as well as *5*,4′-dihydroxy-6,7,8,-trimethoxyflavone (**70**) in *Gardenia lucida* *Roxb*. [91,234,236], antimycobacterial monoterpene indole alkaloids [230], the chalcone butein (**71**) from *H. formicarum* [22] (Table S2) [88,237], and the anthraquinones damnacanthal (**72**) and 1,3-dihydroxy-5-methoxy-2,6-bismethoxymethyl-9,10-anthraquinone [233] (Table S2) [81,229,230,232]. (*E*)-phytol (**73**) is strongly antimycobacterial [232]. The ethanol extract of *M. citrifolia* is weakly active with the FMDV [238] and the methanol extract of *Psychotria serpens* L. with HSV [155]. The essential oil of the latter is strongly bactericidal with *S. aureus* (MIC/MBC: 39/39 µg/mL) [239].

*Order Lamiales:* In the family Acanthaceae, the chloroform extract of the leaves of *Acanthus ebracteatus* Vahl inhibited the growth of *B. cereus*, *S. aureus*, *P. aeruginosa*, and *Proteus vulgaris, C. albicans*, *Aspergillus fumigatus*, and *A. niger* [240]. This true mangrove herb yields the antibacterial 3,5-dimethoxy-4-hydroxy methyl benzoic acid (**74**), (Z)-4-coumaric acid 4-*O*-β-D-glucopyranoside (**75**), and 6-hydroxy-benzoxazolinone (**76**) (Table S2) [241]. The alcohol extract given to ducklings orally at a dose of 2 g/kg/day for 14 days evoked a decrease in serum hepatitis B surface antigen, AST, ALT, and improved the hepatic cytoarchitecture [242]. The ethanol extract of roots inhibited the replication of the NDV, Vaccinia virus, ECMV, and SFV [130]. 

*Avicennia* species (Avicenniaceae) are true mangrove trees yielding broad-spectrum antibacterials [243,244,245] such as the diterpenes excoecarin A (**77**), *ent*-16-hydrozy-3-oxo-13-*epi*-manoyl-oxide (**78**), *ent*-15-hydroxy-labda-8(17), 13*E*-dien-3-one (**79**), which repressed *Rhizopus orizae* and *A. niger* and rhizophorin B with *B. subtilis* (Table S2) [246]. The ethanol extract of *Avicennia alba* L. and *Avicennia officinalis* L. restrained ECMV [130].

*Dolichandrone spathacea* (Burm. f.) Bedd. (Bignoniaceae) is a true mangrove tree yielding the hydroxycinnamic acid glycoside derivatives decaffeoyl acteoside (**80**) and verbascoside (**81**) active against *E. faecalis* (ATCC 1034) and *S. sonnei* (Table S2) [247]. Verbascoside (**81**) very strongly hindered RSV (Table S2) [248]. 

An extract of the stems and leaves of *Myoporum bontioides* (Siebold & Zucc.) A. Gray (Myoporaceae) moderately inhibited the growth of *F. oxysporum*, *Pestalotia mangiferae*, *Thielaviopsis paradoxa*, *Colletotrichum musae*, *Alternaria alternata*, *Mycosphaerella sentina*, and *Sphaceloma fawcettii* [249]. From this plant, the sesquiterpenes myoporumine A (**82**) and B (**83**), (-)-epingaione (**84**), and (–)-dehydroepingaione (**85**) strongly repressed MRSA (Table S2) [250]. (-)-Epingaione is a strong inhibitor of filamentous fungi [249]. Other antifungal principles in this plant are homomonoterpenes (Table S2) [251]

In the Verbenaceae, *Premna odorata* Blanco produces the strong antimycobacterial long chain alkane 1-heneicosyl formate (**86**) (Table S2) [252]. *Essential oil of plants in this family like in the Lamiaceae are often antifungal* [253]. *The methanol extract* *(200 µg/disc)* *developed halos with A. niger and Penicillium cyclopium* [254,255]. 

*Order Solanales*: The ethanol extract of the flowers of *Ipomoea pes-caprae* (L.) R. Br. (Convolvulaceae) inhibited the growth of *S. aureus*, *B. subtilis*, *Streptococcus mutans*, *P. vulgaris*, *K. pneumoniae*, *E. coli*, *A. flavus*, *A. niger*, and *Penicillium* sp [256]. The methanol extract of the leaves of *Solanum viride* R. Br. (Solanaceae) was weakly active against *S. aureus* and *C. albicans* [257].

#### 2.4.11. Clade Campanulids

The ethanol extract of the roots of *Pluchea indica* (L.) Less. (Asteraceae) repressed *E. coli*, *B. cereus*, *Pseudomonas fluorescens*, *S. aureus*, and *S. typhimurium* [258]. The aqueous extract of leaves inhibited HIV-1 [259]. The aqueous extract of berries of *Scaevola taccada* (Gaertn.) Roxb. (Goodeniaceae) restrained HIV-1 [259]. The methanol extract of leaves (500 µg/well) evoked halos with *V. cholerae*, *K. pneumoniae*, *S. typhi*, *S. sonnei*, *F. oxysporum*, *Fusarium solani*, *Rhizoctonia solani*, and *Odium monilioides* [260]. This coastal shrub yields the strong antifungal furanocoumarin scataccanol (**87**) as well as 4-formylsyringol (**88**) (Table S2) [261]. 

## 3. Antimicrobial Extracts and Compounds from Mangrove and Mangrove-Associated Plants with the Potential to Be Used for Shrimp Farming

According to Kuete (2010), crude extracts with MIC values less than 100 µg/mL are antimicrobial [10]. Here, we define a very strongly active extract with a MIC value below 10 µg/mL. An isolated compound is defined as very strongly active for a MIC value below or equal to 1 µg/mL (as well as less than 1 µg/thin layer chromatography), strongly antibacterial (or antifungal) for a MIC value above 1 µg/mL and equal to or below 50 µg/mL, moderately antibacterial (or antifungal) for a MIC from 50 and below 100 µg/mL, weakly antibacterial (or antifungal) for a MIC from 100 and below 500 µg/mL, very weakly antibacterial (or antifungal) for a MIC ranging from 500 to below 2500 µg/mL, and inactive for a MIC value above 2500 µg/mL. 

For antiviral principles, we suggest that a compound with an IC_50_ value below or equal to 1 µg/mL is very strongly active, for an IC_50_ value above 1 and equal to or below 20 µg/mL strongly antiviral, for an IC_50_ above 20 and below or equal to 100 µg/mL moderately antiviral, for an IC_50_ above 100 and below or equal to 500 µg/mL weakly antiviral, for an IC_50_ ranging from above 500 to below or equal to 2500 µg/mL very weakly antiviral, and inactive with an IC_50_ value above 2500 µg/mL. 

Using these criteria, the strongest antimicrobial extracts from mangrove and mangrove-associated plants that could be of value for shrimp farming are from *C. inophyllum* (*S. aureus*, *T. rubrum*) [63], *T. catappa* (*E. faecalis*) [129]., *C. manghas* (*E. coli*, *P. aeruginosa*, VSV) [17,220], and *C. odollam* (HSV) [223].

**Figure 1:**
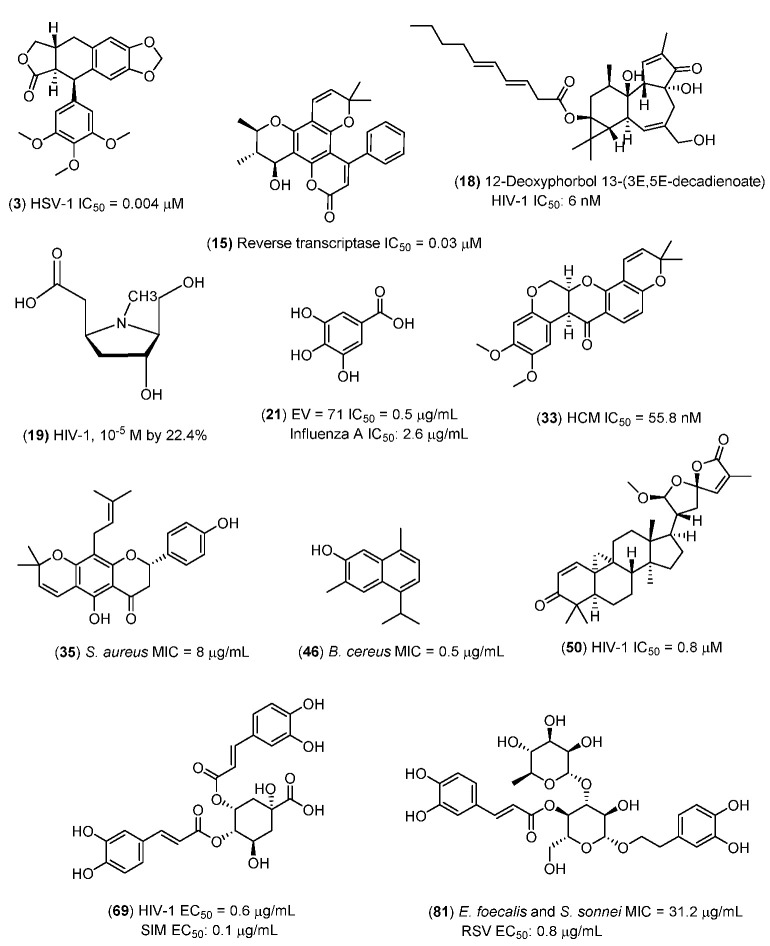
Natural products from mangrove plants with very strong antimicrobial activities.

The strongest antimicrobial principles identified from the mangrove and mangrove-associated plants that could be of value for shrimp farming are as follows (Figure 1):

(i) Antibacterial: Lupinifolin (**35**) (Gram-positive and Gram-negative) [116]; 7-hydroxycadalene (**46)** [152].

(ii) Antifungal: Lupinifolin (**35**) (Yeasts) [116].

(iii) Antiviral: Naringenin (**9**) [50], verbascoside (**81**) [248], inophyllum B (**15**) [61], 12-deoxyphorbol 13-(3*E*,5*E*-decadienoate) (**18**) [69], 5*β*-carboxymethyl-3*α*-hydroxy-2*β*-hydroxymethyl-1- methylpyrrolidine (**19**) [70], deguelin *(***33***)* [117], deoxypodophyllotoxin (**3**) [25,116] (9*R*,10*R*, 23*R*)-21,23:23,27-diepoxycycloarta-1,24-diene-3,27-dione (**49**) [156], gallic acid (**21**) [83], and 4,5-di-*O*-caffeoylquinic acid (**69**).

(iv) We note that most of these principles are hydrophilic or amphiphilic (Figure 1). 

## 4. Spectrum of Activity of Antimicrobial Extracts and Principles from Mangrove and Mangrove-Associated Plants

The following observations can be made:

(i) No reports on the only lycopod associated with mangrove are available.
(ii) Of the 26 ferns, nine had antibacterial effects and six are antifungal, and no antiviral activities were reported. The only antimicrobial principle from ferns thus far is the strong antibacterial (Gram-positive) stenopalustroside A [13] (Table 1).
(iii) The cycad associated with mangroves has antibacterial effects.
(iv) No reports on the only pine tree associated with mangrove are available.
(v) Of the 51 monocots, 11 displayed antimicrobial effects, of which eight had antibacterial activity, six with antiviral activity, and none reported with antiviral properties. Active principles isolated were phenolics such as the flavanol naringenin (**9**) in the Pandanaceae, antibacterial, antifungal, and antiviral orchidaceous phenanthrenes as well as the flavones and antifungal hydroxycinnamic acid of Poaceae (Table 2)
(vi) Of the 207 dicots, 92 had antimicrobial effects including 78 antibacterial, 39 antifungal, and 25 antiviral effects. A total of about 80 antimicrobial principles were isolated (Table S2).
(vii) Aqueous and organic polar extracts of plants from the mangrove presented activity against Gram-positive and Gram-negative bacteria, filamentous fungi and yeasts, enveloped and non-enveloped viruses, DNA, and RNA viruses (Table S2).
(viii) The extract of *P. pinnata* [262] and gallic acid (**21**) abounds notably in the true mangrove trees *Rhizophora apiculata* Bl. and *Aegiceras corniculatum* (L.) Blanco is a protected shrimp against WSSV [84] as well as an aqueous extract of the true mangrove tree *C. tagal* (Perr.) C.B. Rob. [263]

## 5. Medicinal Use of Mangrove and Mangrove-Associated Plants

One could suggest the use of medicinal plants as a more sustainable alternative to chemotherapy in paenid aquaculture. Therefore, the possible beneficial effect of mangrove and mangrove-associated plants for the sanitation of shrimp farms is reinforced by the observation that 85 plants were used for the treatment of infectious diseases including mainly diarrhea, dysentery, and wounds [264,265,266,267,268,269,270,271,272,273,274,275,276,277,278,279,280,281,282,283,284,285,286,287,288,289,290,291,292,293,294] (Table S1). The pharmacological effect of these plants involves active principles that are potentially able to act on paenids, which could be examined further.

## 6. Mangrove and Mangrove-Associate Plants as Remediation of Shrimp Farming?

Shrimp and prawn farms are regularly affected by (+)-RNA viruses such as the Taura syndrome virus, Yellow head virus, and Gill-associated virus as well as DNA viruses (WSSV, Monodon Baculovirus) and Gram-negative bacteria such as *Hepatobacterium penaei* and *Vibrio* spp. [295]. Synthetic drugs are being used in an attempt to evade economic losses but threaten the environment and contribute to the selection of multidrug-resistant pathogenic microorganisms. Being able to produce antimicrobial principles (some of them water soluble like ellagic acid), mangrove and mangrove-associated plants could be used as a source of natural agents and/or afford ecological systems to combat the infections with shrimps and prawns. Polar organic and aqueous extracts of most mangrove and mangrove-associated plants exhibit broad-spectrum antibacterial, antifungal, or antiviral properties in vitro, suggesting that antimicrobial secondary metabolites from plants and plant litter in the sea and brackish waters could afford some control against the overgrowth of pathogenic microbes. Of note, *P. pinnata* ethanol extract of leaves given to Penaeus monodon as part of feed at the dose of 300 μg/g of body weight/day evoked some levels of protection against WSSV [262]. Gallic acid (**21**), which abounds notably in the true mangrove trees *R. apiculata* and *A. corniculatum* is strongly antiviral and protected shrimps against WSSV [84]. Gallic acid (**21**) may, at least in part, account for the fact the aqueous extract of the true mangrove tree *C. tagal* given at the dose of 10% of the body weight, twice a day, protected shrimps against WSSV [263]. Furthermore, gallic acid (**21**) decreases microbial proliferation in mangrove soil [296] as well as the growth of microalgae [297], which contribute to a decreased production in shrimp aquaculture [298], at least in part, to the alteration in the shrimp’s immune system [299]. The control of pathogenic bacteria may have some beneficial effects for the symbiotic bacteria of shrimp against pathogenic microorganisms [300]. Furthermore, phenolic acids from mangrove and mangrove-associated plants could, by chelation, protect shrimps against toxic metals including cadmium [301,302]. Therefore, it is possible to extend the protective effect of mangrove and mangrove-associated plants to fisheries and crab farming, the latter being affected by *Vibrio* species [165]. Another interesting feature of mangrove plants is that they are a host for microorganisms for Actinomyces producing antibacterial principles [303].

## 7. Conclusions

Plants from the Mangroves of Asia and the Pacific produce a vast array of antimicrobial secondary metabolites that could be further examined for their possible development into medications to control microbial outbreaks in aquaculture. In parallel, growing plant mangroves in aquacultures and promoting mangrove-associated aquaculture could be beneficial. The rise in the global population and the imperative to supply shrimps, prawns, crabs, and fish globally requires the preservation of mangroves.

## Figures and Tables

**Table 1:** Ferns and cycads from the mangroves, tidal rivers, and the seashores of Asia and the Pacific with antibacterial and/or antifungal activity.

FAMILY Genus, Species	Extract		Secondary Metabolite Identified
	Antibacterial	Antifungal	
SUBCLASS POLYPODIIDAE			*Antibacterial:* Stenopalustroside A (1), *S. epidermidis* (MIC = 2 µg/mL) [13].
BLECHNACEAE *Stenochlaena palustris* (Burm. f.) Bedd.	+	+	
NEPHROLEPIDACEAE *Nephrolepis biserrata* (Sw.) Schott	+	+	
POLYPODIACEAE *Drynaria quercifolia* (L.) J. Sm.	+	+	
*Drymoglossum piloselloides* (L.) Presl.	+	+	
*Microsorum punctatum* (L.) Copel.	+	+	
*Platycerium coronarium (O.F. Müll.) Desv.*	+		
*Pyrrosia piloselloides* (L.) M.G. Price)	+	+	
PTERIDACEAE *Acrostichum aureum* L.	+	+	
*Acrostichum speciosum* Willd.	+		
SUBCLASS CYCADIIDAE			
CYCADACEAE *Cycas rumphii* Miq.	+		

Bold: true mangrove plants [3]. +: Activity of extract(s) reported in the literature.

**Table 2:** Monocots from the mangroves, tidal rivers, and the seashores of the Asia and the Pacific with antibacterial, antifungal, and/or antiviral activity.

FAMILY Genus, Species (Synonym)	Extracts			Antimicrobial Principle(s)
	Antibacterial	Antifungal	Antiviral	
ARACEAE *Lasia spinosa* (L.) Thwaites	+	+		*Antibacterial:* Meridinol (7) (100 µg/disc) [47]. *Antifungal:* Meridinol (7) (100 µg/disc) [47].
ARECACEAE *Phoenix paludosa* Roxb.	+	+		*Antibacterial:*3′-Acetoxy-6,7-dimetoxy-4′ (2″,3″,4″,6″- tetraacetylglucopyranosyl)flavone (10), *P. aeruginosa*, *E. coli*, *S flexneri*, MIC= 8, 4, and 8 µg/mL, respectively [47]. Tricin (8), *P. aeruginosa*, *E. coli*, *S. flexneri,* MIC = 4, 2, and 2 µg/mL, respectively [47]. Cinnamic acid (11), *P. aeruginosa*, *E. coli*, *S. flexneri*, MIC = 64, 16, and 16µg/mL, respectively [47]. *Antifungal:* 3′-Acetoxy-6,7-dimetoxy-4′ (2″,3″,4″,6″- tetraacetylglucopyranosyl)flavone (10): *C. neoformans*, *C. albicans*, *C. parapsilosis*, MIC: of 16, 8, and 8 µg/mL, respectively [47]. Tricin (8), *C.* neoformans, *C. albicans*, *C. parapsilosis,* MIC = 8, 4, and 4 µg/mL, respectively [47]. Cinnamic acid (11), *C.* *neoformans*, *C. albicans*, *C. parapsilosis*, MIC = 64, 32, and 32 µg/mL, respectively [47].
*Saribus rotundifolius* (Lam.) Bl.	+	+		
CYPERACEAE *Cyperus scariosus* R. Br.		+		
*Eleocharis dulcis* (Burm. f.) Trin. ex Hensch	+			
*Rhynchospora corymbosa* (L.) Britton				
FLAGELLARIACEAE *Flagellaria indica* L.	+	+		*Antiviral:* Tricin (8), IVA, IC_50_ = 4.6 µM, HIV-1, IC_50_ = 14.4 µg/mL [49].
ORCHIDACEAE *Aerides odoratum* Reinw. ex Bl.	+			
*Cymbidium finlaysonianum* Wall. ex Lindl.		+		*Antibacterial:* Batatasin III (4), *S. aureus*, *B. subtilis,* MRSA, MIC = 250, 500, and 500 µg/mL, respectively [43]. *Antifungal:* Batatasin III (4), *A. brassicicola*, *P. parasitica*, *C. capsici*, *B. oryzae*, *D.medusaea, C. paradoxa moreau*, *E.turcicum*, *P. theae*, *A. citri* [44]. *Antiviral:* Batatasin III (4), HSV-1, HSV-2, IC_50_ = 341.5 and 384.2 µM, respectively [45]. Gigantol (5), HSV-1 and HSV-2, IC_50_ = 304.1 and 319.3 µM, respectively [45].
*Dendrobium moschatum* (Buch. -Ham.) Sw.				*Antibacterial:* Moscatin (6), *V. parahemolyticus*, *S. gallinarum*, *S. aureus*, *S. agalactiae*, *E. faecalis*, *B. subtilis*, *R. anatipestifer*, MIC = 96, 72, 72, 48, 96, 72, and 72 µg/mL, respectively [46].
PANDANACEAE *Nypa fruticans* Wurmb.	+			*Antiviral:* Naringenin (9), SARS-CoV, 65.2 µM [51]; YFV, EC_50_: 0.001 M; ZKV [50]
*Pandanus tectorius* Parkinson				
POACEAE *Phragmites vallatoria* Veldkamp	+	+		
RUPPIACEAE *Ruppia maritima* L.	+			

+: Activity of extract(s) reported in the literature.

## Data Availability

Not applicable.

## Supplementary Materials

The following supporting information can be downloaded at: https://www.mdpi.com/article/10.3390/md20100643/s1, Figure S1: Natural products from the plants of the mangroves, tidal rivers, and the seashores of Asia and the Pacific; Table S1: Plants from the mangroves, tidal rivers, and the seashores of Asia and the Pacific, Table S2: Antimicrobial activity of extracts and isolates from the Dicotyledons from the mangroves, tidal rivers, and the seashores of Asia and the Pacific.
