# Supplementary material for: Antimicrobial Secondary Metabolites from the Mangrove Plants of Asia and the Pacific
Source: Mar Drugs. 2022 Oct 15;20(10):643. doi: 10.3390/md20100643 (PMC9605323; doi:10.3390/md20100643)
Supplement: Supplementary file 1 [file marinedrugs-20-00643-s001.zip › Figure S1.pdf]

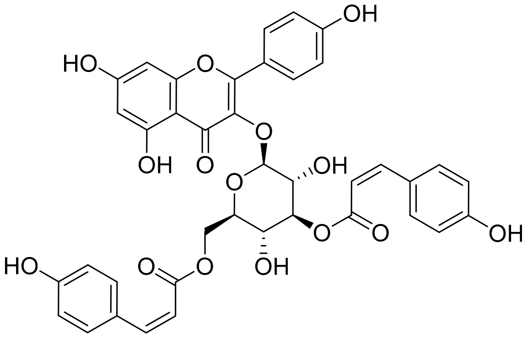
(**1**)

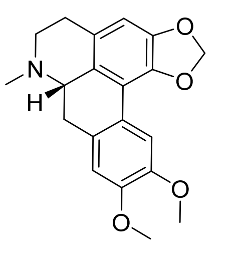
(**2**)

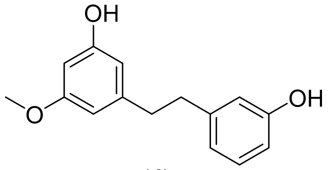
(**4**)

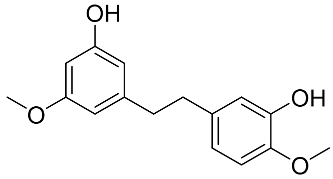
(**5**)

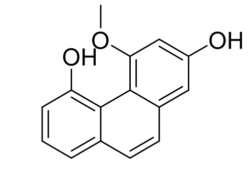
(**6**)

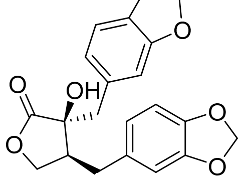
(**7**)

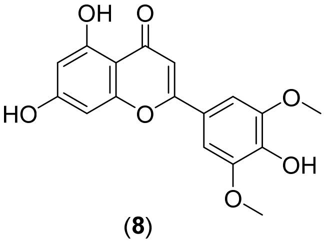
(**8**)

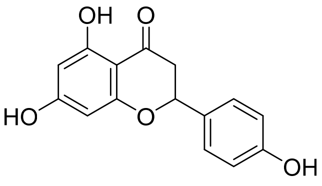
(**9**)

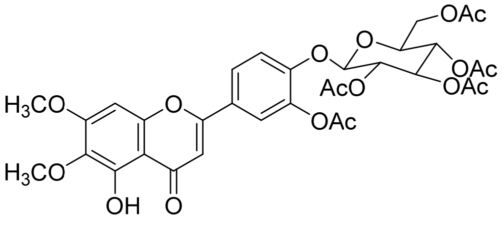
(**10**)

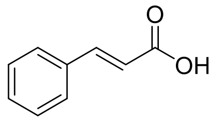
(**11**)

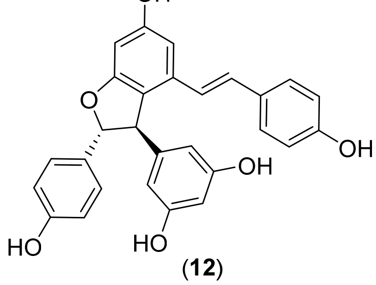
(**12**)

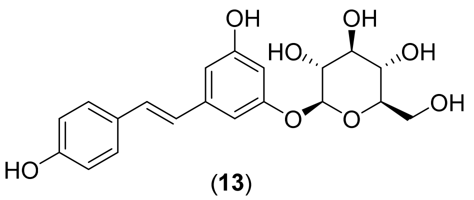
(**13**)

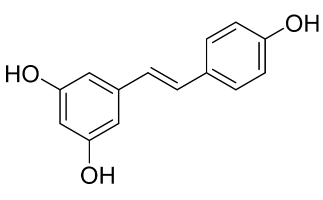
(**14**)

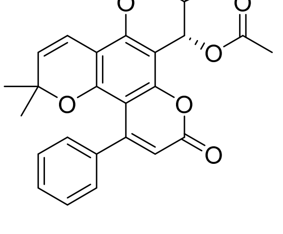
(**16**)

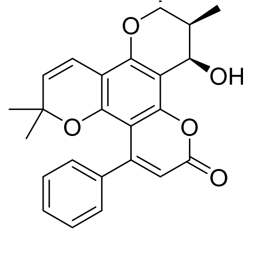
(**17**)

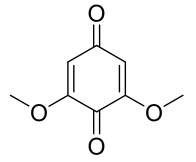

(**20**)

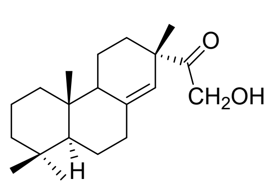

(**22**)

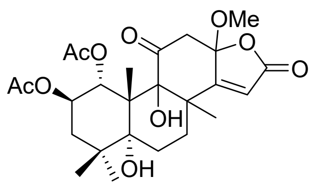

(**23**)

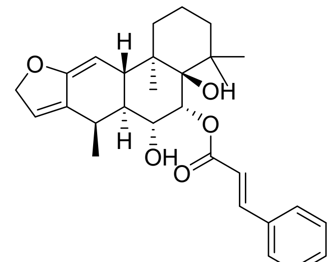

(**24**)

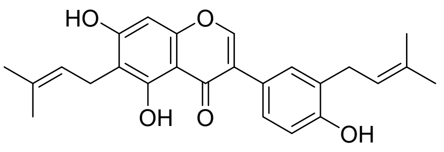

(**25**)

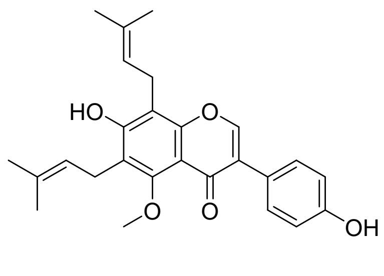

(**26**)

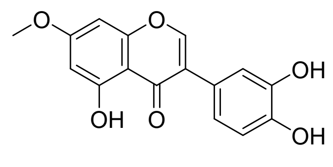

(**27**)

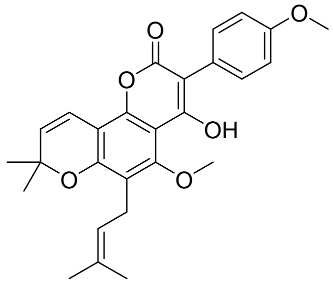

(**28**)

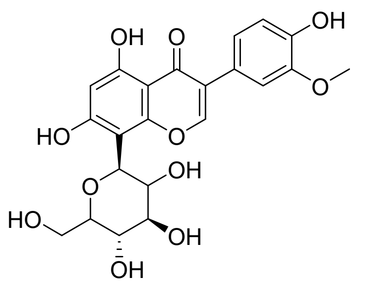

(**29**)

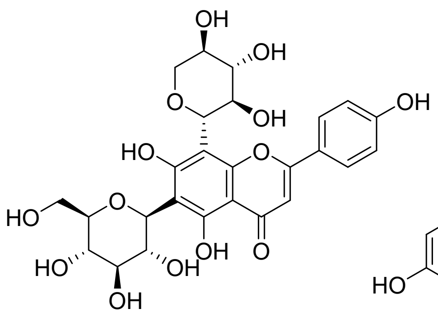

(**30**)

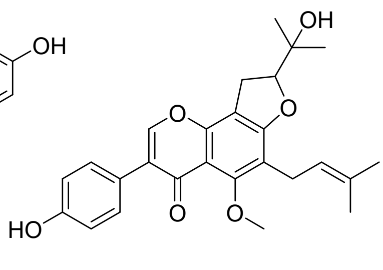

(**31**)

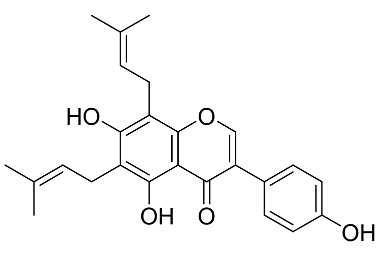

(**32**)

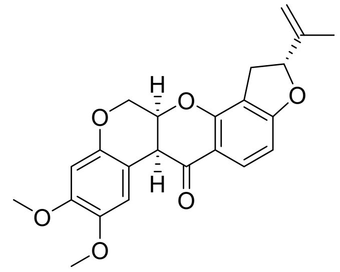

(**34**)

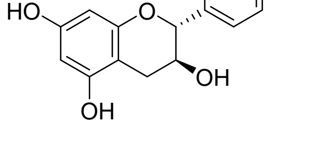

(**36**)

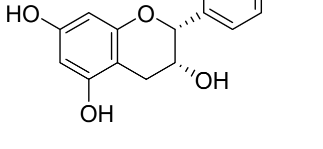

(**37**)

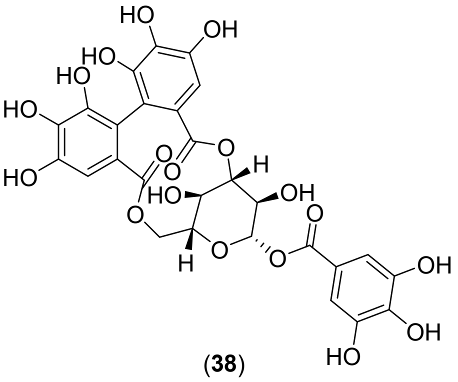

(**38**)

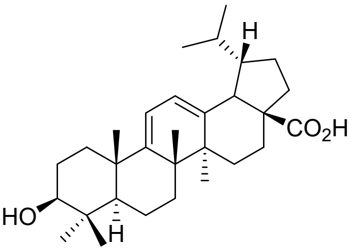

(**39**)

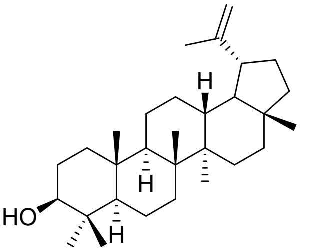

(**40**)

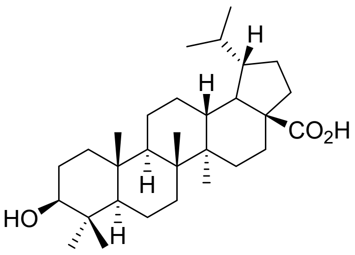

(**41**)

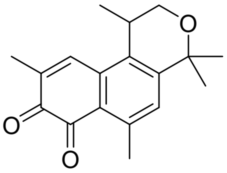

(**42**)

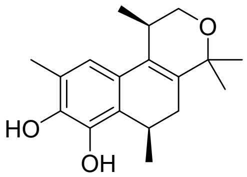

(**43**)

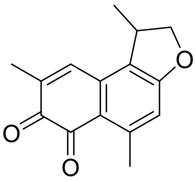

(**44**)

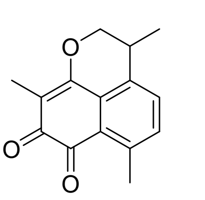

(**45**)

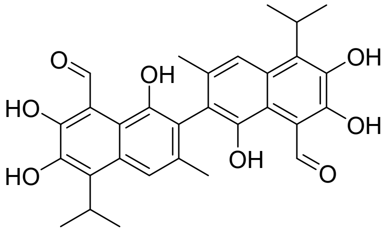

(**47**)

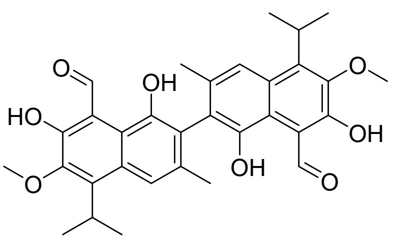

(**48**)

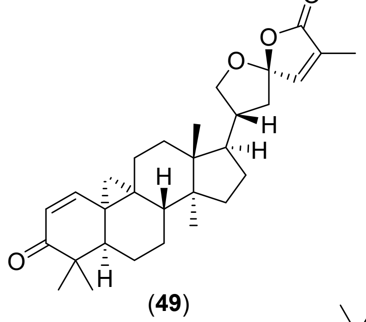

(**49**)

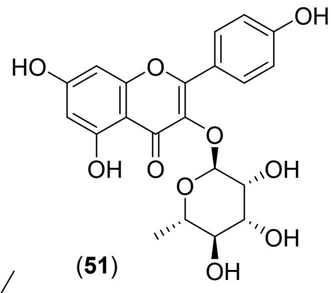

(**51**)

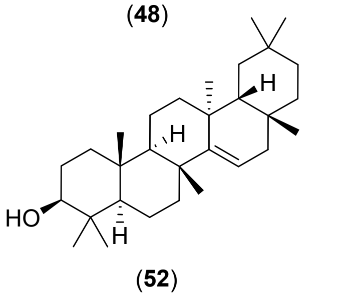

(**52**)

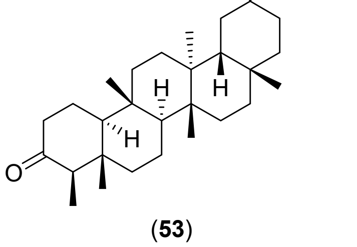

(**53**)

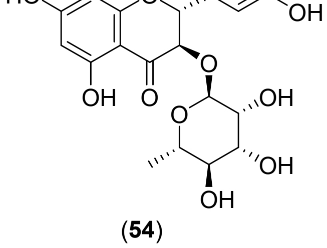

(**54**)

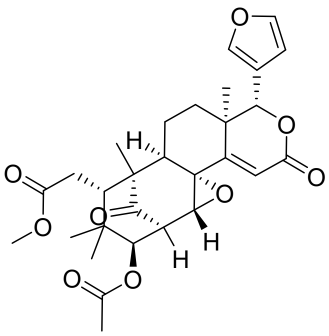

(**55**)

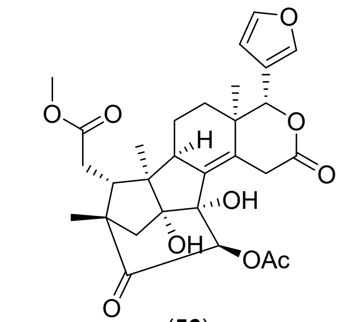

(**56**)

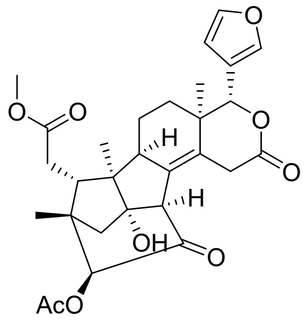

(**57**)

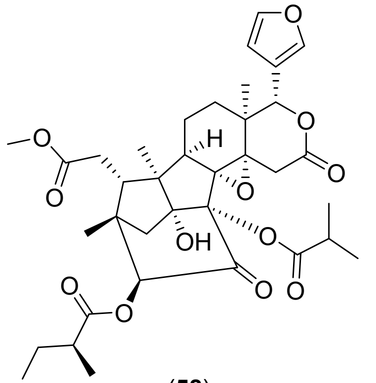

(**58**)

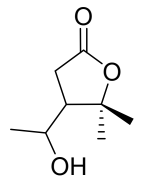

(**59**)

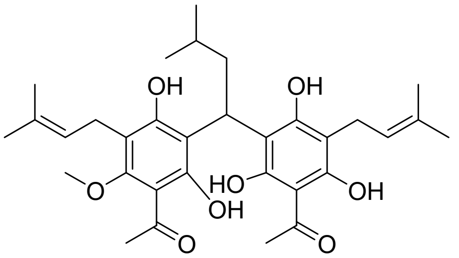

(**60**)

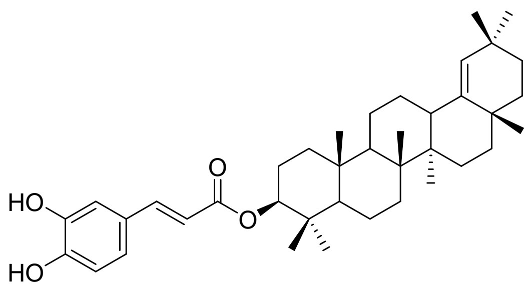

(**61**)

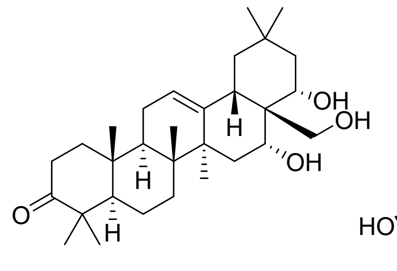

(**62**)

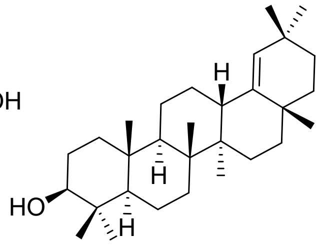

(**63**)

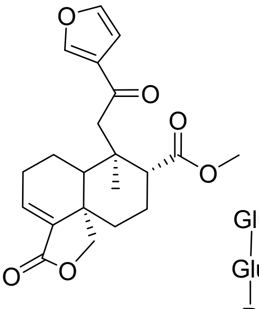

(**64**)

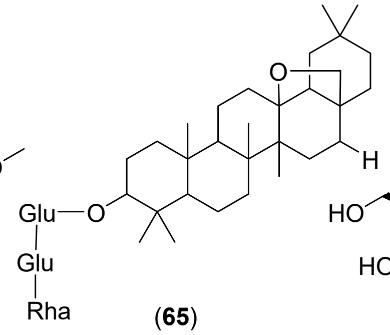

(**65**)

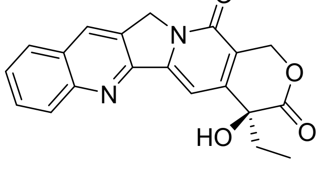

(**66**)

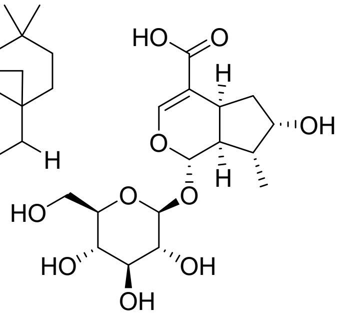

(**67**)

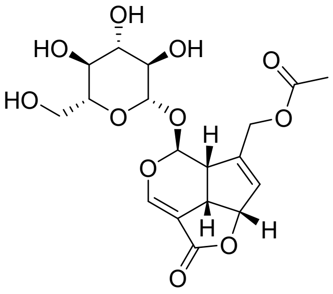

(**68**)

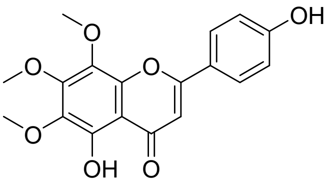

(**70**)

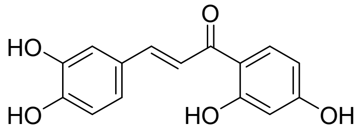

(**71**)

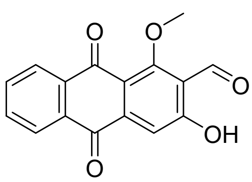

(**72**)

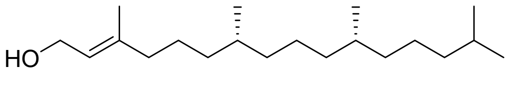

(**73**)

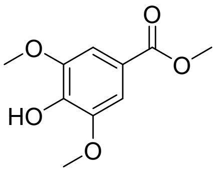

(**74**)

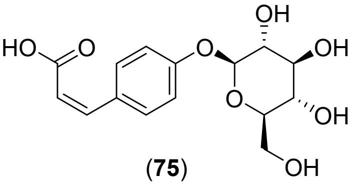

(**75**)

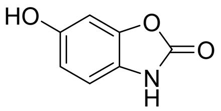

(**76**)

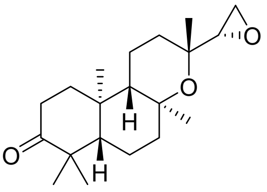

(**77**)

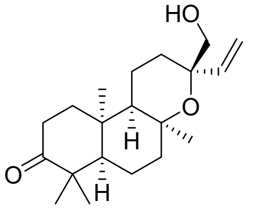

(**78**)

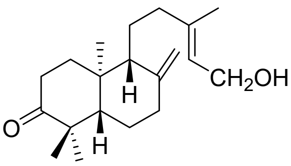

(**79**)

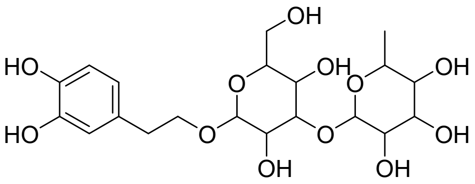

(**80**)

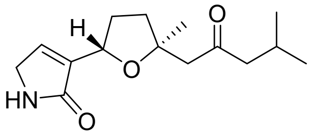

(**82**)

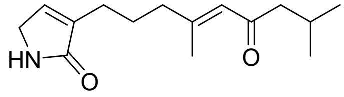

(**83**)

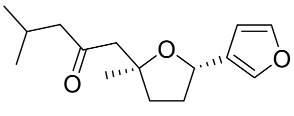

(**84**)

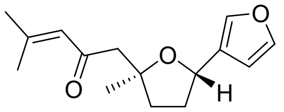

(**85**)

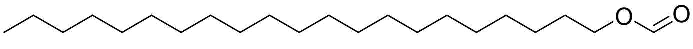

(**86**)

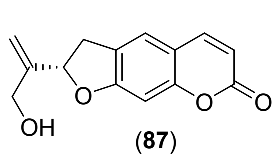

(**87**)

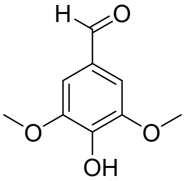

(**88**)

Figure S1. Natural products from the plants of the mangroves, tidal rivers, and the seashores of Asia and the Pacific
