# Supplementary material for: Antimicrobial Secondary Metabolites from the Mangrove Plants of Asia and the Pacific
Source: Mar Drugs. 2022 Oct 15;20(10):643. doi: 10.3390/md20100643 (PMC9605323; doi:10.3390/md20100643)
Supplement: Supplementary file 1 [file marinedrugs-20-00643-s001.zip › Table S1.pdf]

Table S1. Plants from the Mangroves, Tidal Rivers, and the Seashores of Asia and the Pacific

FAMILY<br>Genus, species<br>(synonym)	Distribution	Medicinal use (country) ref.
SUBCLASS LYCOPODIIDAE		
LYCOPODIACEAE<br>*Lycopodium carinatum* Desv. ex Poir<br>(*Phlegmariurus carinatus* (Desv. ex Poir.) Ching).	India, Bangladesh, Southeast Asia, China, Papua, Pacific	
SUBCLASS POLYPODIIDAE		
ASPLENIACEAE<br>*Asplenium macrophyllum* Sw.<br>(*Asplenium capuronii* Tardieu)	India, Bangladesh, Southeast Asia, Papua, Australia, Pacific	
BLECHNACEAE<br>*Stenochlaena palustris* (Burm. f.) Bedd<br>(*Polypodium palustre* Burm. f.,<br>*Pteris scandens* (Willd.) Roxb.)	India, Bangladesh, Southeast Asia, Papua, Australia	
DAVALLIACEAE<br>*Davallia denticulata* (Burm. f.) Mett. ex Kuhn(*Adiantum denticulatum* Burm. f.)	India, Bangladesh, Southeast Asia, Papua, Australia	
Davallia parvula Wall. ex Hook. & Grev.<br>(*Humata parvula* (Wall. ex Hook. & Grev.) Mett.)	Malaysia, Indonesia, Papua	
Davallia angustata Wall. ex Hook. & Grev.<br>(*Humata angustata* (Wall. ex Hook. & Grev.) J. Sm.,*Pachypleura angustata* (Wall. ex Hook. & Grev.) J. Sm.)	Thailand, Vietnam, Malaysia, Indonesia	
DRYOPTERIDACEAE<br>*Elaphoglossum amblyphyllum* P.R. Bell.	Malaysia, Indonesia	
HYMENOPHYLLACEAE<br>*Hymenophyllum holochilum* (Bosch) C. Chr.(*Didymoglossum holochilum* Bosch)	India, Bangladesh, Southeast Asia, Papua, Australia, Pacific	
NEPHROLEPIDACEAE<br>*Nephrolepis acutifolia* (Desv.) Christ	Southeast Asia, Papua, Australia, Pacific	Diarrhoea (Indonesia) [264]
Nephrolepis biserrata (Sw.) Schott (*Aspidium biserratum* Sw.)	India, Bangladesh, Southeast Asia, Papua, Australia, Pacific	Wounds (Indonesia) [264]

Nephrolepis falcata (Cav.) C. Chr.<br>(*Tectaria falcata* Cav.)	the Philippines	
POLYPODIACEAE<br>*Ctenopteris moultoni* (Copel.) C. Christ. & Tardieu<br>(*Polypodium moultonii* Copel.,<br>*Ctenopterella blechnoides* (Grev.) Parris)	Thailand, Malaysia, Indonesia	
Drynaria rigidula (Sw.) Bedd.<br>(*Polypodium rigidulum* Sw.)	Malaysia, Thailand, Indonesia, Papua	Gonorrhoea (Indonesia) [279]
Drynaria quercifolia (L.) J. Sm.<br>(*Polypodium quercifolium* L.)	India, Bangladesh, Malaysia, Thailand, Indonesia, Papua, Australia	Cough (India) [293]
Drynaria sparsisora (Desv.) T. Moore<br>(*Polypodium sparsisorum* Desv.)	Malaysia, Thailand, Indonesia, Papua, Australia	
Drymoglossum piloselloides (L.) Presl.<br>(*Pteris piloselloides* L., *Pyrrosia piloselloides* (L.) M.G. Price)	India, Bangladesh, Southeast Asia, China, Japan, Papua	Smallpox (the Philippines) [278]
Loxogramme involuta (D. Don) C. Presl<br>(*Grammitis involuta* D. Don,<br>*Gymnogramma involuta* (D. Don) Hook.)	Bangladesh, Myanmar, Thailand, Cambodia, Vietnam, China	
Microsorum punctatum (L.) Copel.<br>(*Acrostichum punctatum* L.)	India, Bangladesh, Southeast Asia, Papua, Australia	
Myrmecophila sinuosa (Hook.) Nakai ex H. Itô<br>(*Lecanopteris sinuosa* (Wall. ex Hook.) Copel.,<br>*Polypodium sinuosum* Hook.)	Southeast Asia, Papua, Australia	
Photinopteris speciosa (Bl.) C. Presl<br>(*Drynaria speciosa* (Bl.) Christenh.,<br>*Photinopteris acuminata* (Willd.) C.V. Morton)	Southeast Asia, China	
Phymatosorus scolopendria (Burm. f.) Pic. Serm.<br>(*Microsorum scolopendria* (Burm.f.) Copel.,<br>*Phymatodes scolopendria* (Burm. f.) Ching,<br>*Polypodium scolopendria* Burm. f.)	Southeast Asia, Papua, Australia, Pacific	Wounds (Samoa) [278]
Platycerium coronarium (O.F. Müll.) Desv.<br>(*Osmunda coronaria* O.F. Müll.)	Southeast Asia	

Pyrrosia lanceolata (L.) Farw.<br>(*Acrostichum lanceolatum* L.)<br><br>*Pyrrosia longifolia* (Burm. f.) C.V. Morton<br>(*Acrostichum longifolium* Burm. f.<br><br>PTERIDACEAE<br>***Acrostichum aureum* L.**<br>(*Acrostichum inaequale* Willd.,<br>*Chrysodium aureum* (L.) Mett.)<br><br>***Acrostichum speciosum* Willd.**<br>(*Acrostichum aureum* var.*speciosum* (Willd.) T. Moore)<br><br>*Vittaria elongata* Sw.<br>(*Haplopteris elongata* (Sw.) E.H. Crane)	Southeast Asia, Papua, Australia, Pacific<br><br><br>Southeast Asia, Papua, Australia Pacific<br><br><br><br>India, Bangladesh, Southeast Asia, Papua, Australia, Pacific<br><br><br><br>Southeast Asia, Papua, Australia<br><br><br>India, Bangladesh, Southeast Asia, China, Papua, Australia	 <br><br><br><br><br><br>Fever (Fiji) [292]
SUBCLASS CYCADIIDAE<br><br>CYCADACEAE<br>*Cycas rumphii* Miq.	Indonesia, Papua, Australia	Wounds (Indonesia) [277]
PODOCARPACEAE<br><br>*Podocarpus polystachyus* R. Br. ex Endl.<br>(*Margbensonia polystachyus* (R. Br. ex Endl.) A.E Bobrov & Melikyan,<br>*Nageia polystachia* (R. Br. ex Endl.) Kuntze)	Thailand, Malaysia, Indonesia, the Philippines	
SUBCLASS MAGNOLIIDAE-MONOCOTS<br><br>ARACEAE<br>*Aglaonema griffithii* Schott<br>(*Aglaodorum griffithii* (Schott) Schott)<br><br>*Cryptocoryne ciliata* (Roxb.) Fisch. ex Schott<br>(*Ambrosina ciliata* Roxb.)<br><br>*Lasia spinosa* (L.) Thwaites<br>(*Dracontium spinosum* L.)<br><br>ARECACEAE<br>*Calamus erinaceus* (Becc.) J. Dransf.<br>(*Daemonorops erinacea* Becc.)<br><br>*Oncosperma tigillarium* (Jack) Ridl.	 <br><br>Thailand, Malaysia, Vietnam, Indonesia<br><br><br>India, Bangladesh, Southeast Asia, Papua<br><br><br>India, Bangladesh, Southeast Asia, China, Papua<br><br><br><br>Southeast Asia<br><br><br>Southeast Asia	 <br><br><br><br><br><br><br><br>Tuberculosis (China) [280]

(*Areca tigillaria* Jack, *Oncosperma filamentosa* Bl.)		
Phoenix paludosa Roxb.	India, Bangladesh, Southeast Asia	
Saribus rotundifolius (Lam.) Bl.<br>(*Corypha rotundifolia* Lam.)	Indonesia, Papua	
CYPERACEAE		
Baumea juncea (R. Br.) Palla<br>(*Cladium junceum* R. Br.)	Australia, New Caledonia	
Cyperus elatus L.<br>(*Cyperus distans* L.f.)	India, Bangladesh, Southeast Asia, Papua	
Cyperus javanicus Houtt.<br>(*Cyperus pennatus* Lam.)	India, Bangladesh, Southeast Asia, Papua, Pacific	
Cyperus malaccensis Lam.<br>(*Cyperus fortunei* Steud.)	India, Bangladesh, Southeast Asia, Papua	
Cyperus stolonifer Retz.<br>(*Cyperus tuberosus* Bojer)	India, Bangladesh, Southeast Asia, Papua, Australia, Pacific	
Cyperus tagetum Roxb.	India	
Cyperus scariosus R. Br.	Papua, Australia	
Eleocharis dulcis (Burm. f.) Trin. ex Hensch.<br>(*Scirpus spiralis* Rottb.)	India, Bangladesh, Southeast Asia, Papua, Australia, Pacific	
Fimbristylis cymosa R. Br.<br>(*Fimbristylis spathacea* Roth)	India, Bangladesh, Southeast Asia, Papua, Australia	
Fimbristylis ferruginea (L.) Vahl<br>(*Scirpus debilis* Lam., *Scirpus ferrugineus* L.)	India, Bangladesh, Southeast Asia, Papua, Australia, Pacific	
Fimbristylis littoralis Gaudich.	India, Bangladesh, Southeast Asia, Papua, Australia, Pacific	
Fimbristylis polytrichoides (Retz.) R.Br.<br>(*Scirpus polytrichoides* Retz.)	India, Bangladesh, Southeast Asia, Papua, Australia	
Fimbristylis subspicata Nees	Japan, Vietnam	
Rhynchospora corymbosa (L.) Britton	India, Bangladesh, Southeast Asia, Papua, Australia	

(*Scirpus corymbosus* L.)		
Scirpus grossus L.f.<br>(*Actinoscirpus grossus* (L. f.) Goetgh. & D.A. Simpson)	India, Bangladesh, Southeast Asia, Papua, Australia, Pacific	Leucorrhea (Bangladesh) [284]
Scirpus kimsonensis N.K. Khoi	Vietnam	
FLAGELLARIACEAE<br>*Flagellaria indica* L.<br>(*Flagellaria minor* Bl., *Flagellaria philippinensis* Elmer)	India, Bangladesh, Southeast Asia, Papua, Australia, Pacific	
JUNCAGINACEAE<br>*Triglochin striata* Ruiz & Pavon<br>(*Triglochin decipiens* R. Br., *Triglochin decipiens* R. Br., *Triglochin montevidensis* Spreng.)	Australia	
ORCHIDACEAE<br>*Aerides odoratum* Reinw. ex Bl.	Southeast Asia, China	Pneumonia (Bangladesh) [283]
Bulbophyllum xylocarpi J.J. Sm.	Southeast Asia, China	
Cymbidium finlaysonianum Wall. ex Lindl.<br>(*Cymbidium aloifolium* (L.) Sw., *Cymbidium pendulum* (Roxb.) Sw.,*Cymbidium pendulum* var. *brevilabre* Lindl.,*Cymbidium tricolor* Miq.)	Southeast Asia	
Dendrobium aloefolium (Bl.) Rchb.f.	Southeast Asia, Papua	
Dendrobium moschatum (Buch.-Ham.) Sw.<br>(*Epidendrum moschatum* Buch.-Ham.)	India, Bangladesh, Myanmar, Cambodia, Laos, China, Laos, Thailand, Vietnam	Otitis (India) [282]
Dendrobium pachyphyllum (Kuntze) Bakh. f.<br>(*Callista pachyphylla* Kuntze)	Southeast Asia	
Dendrobium subulatum (Bl.) Lindl.<br>(*Onychium subulatum* Bl.)	Thailand, Malaysia, Indonesia	
Dockrillia teretifolia (R.Br.) Brieger<br>(*Dendrobium teretifolium* R. Br.)	Indonesia, Papua, Australia	
Oberonia iridifolia Lindl.<br>(*Oberonia ensiformis* (Sm.) Lindl..)	India, Bangladesh, Southeast Asia	

Oberonia anceps Lindl.<br>(*Oberonia lycopodioides* (J.Koenig) Ormerod)	Southeast Asia	Boils(Indonesia) [268]
Oberonia laeta J.J. Sm.	Malaysia, Indonesia	
***Oberonia rhizophoreti* Schltr.**<br>(*Oberonia marina* J.B.Comber)	Malaysia, Indonesia	
PANDANACEAE		
Pandanus affinis Kurz<br>(*Benstonea affinis* (Kurz) Callm. & Buerki)	Vietnam, Malaysia, Indonesia, the Philippines	
Pandanus klossii Ridl.	Malaysia	
***Nypa fruticans* Wurmb.**<br>(*Cocos nypa* Lour., *Nipa litoralis* Blanco)	India, Bangladesh, Southeast Asia, Papua, Australia	
Pandanus tectorius Parkinson	Indonesia, The Philippines, Papua, Australia, Pacific	Boils (Pacific) [281]
Pandanus tonkinensis B.C Stone	Vietnam	
POACEAE		
Fimbristylis cymosa R.Br.	India, Bangladesh, Southeast Asia, Papua, Australia, Pacific	Dysentery (India) [275]
Fimbristylis ferruginea (L.) Vahl<br>(*Scirpus ferrugineus* L.,<br>*Fimbristilis sieberiana* Kunth)	India, Bangladesh, Southeast Asia, Papua, Australia, Pacific	
Fimbristylis polytrichoides (Retz.) Vahl<br>(*Scirpus polytrichoides* Retz.)	India, Bangladesh, Southeast Asia, China, Papua, Australia, Pacific	
Fimbristylis sericea R.Br.<br>(*Scirpus sericeus* Poir.)	India, Bangladesh, Southeast Asia, China, Japan, Australia, Pacific	
Leptochloa fusca (L.) Kunth<br>(*Diplachne fusca* (L.) P.Beauv. ex Roem. & Schult.,<br>*Diplachne malabarica* (L.) Merr., *Festuca fusca* L.,<br>*Panicum malabaricum* (L.) Merr.)	India, Bangladesh, Southeast Asia, China, Japan, Papua, Australia, Pacific	
Paspalum vaginatum Sw.<br>(*Panicum littorale* (R.Br.) Kuntze, *Panicum vaginatum* Nees<br>*Paspalum dissectum* (L.) L., *Digitaria foliosa* Lag.)	Pacific	
Phragmites vallatoria Veldkamp		

(*Phragmites karka* (Retz.) Trin. ex Steud.)	India, Bangladesh, Southeast Asia, China, Japan, Papua, Australia, Pacific	
Sporobolus virginicus (L.) Kunth<br>(*Agrostis virginica* L.)	India, Bangladesh, Southeast Asia, China, Japan, Papua, Australia, Pacific	
RUPPIACEAE<br>*Ruppia maritima* L.	India, Bangladesh, Myanmar, Thailand, Malaysia Australia	
TACCACEAE<br>*Tacca leontopetaloides* (L.) Kuntze<br>(*Leontice leontopetaloides* L.)	India, Bangladesh, Myanmar, Thailand, Malaysia, Indonesia, Papua, Australia	
SUBCLASS MAGNOLIIDAE-DICOTS		
ACANTHACEAE<br>***Acanthus ebracteatus* Vahl**<br>(*Acanthus ilicifolius* L.<br>var. *ebracteatus* (Vahl) Benoist,<br>*Dilivaria ebracteata* (Vahl) Persoon.)	India, Myanmar, Cambodia, Thailand, Vietnam, Malaysia, Indonesia, Papua, Australia, Pacific	Otitis (Palau)[268]
***Acanthus ilicifolius* L.**<br>(*Acanthus xiamenensis* R.T. Zhang,<br>*Dilivaria ilicifolia* (L.) Juss.)	Tropical Asia Pacific	Fever (India)[268]
***Acanthus volubilis* Wall.**<br>(*Dilivaria volubilis* (Wall.) Nees)	India, Bangladesh, Myanmar, Thailand, Malaysia	Boils (India)[268]
Hygrophila phlomoides Nees	Bangladesh, Myanmar, Thailand, Vietnam, Malaysia, the Philippines	Sores (Myanmar)[268]
AEGICERATACEAE<br>***Aegiceras corniculatum* (L.) Blanco**	India, Bangladesh, Southeast Asia, Papua, Australia	Otitis (India)[268]
***Aegiceras floridum* Roemer & Schultes**	the Philippines, Indonesia, Papua	
AIZOACEAE<br>*Sesuvium portulacastrum* (L.) L.<br>(*Portulaca portulacastrum* L.)	Tropical Asia, Pacific	Wounds(India)[268]
ANACARDIACEAE<br>*Gluta velutina* Bl.	Bangladesh, Myanmar, Thailand, Malaysia, Indonesia, Vietnam	

ANNONACEAE		
Annona palustris L.	India, China, Australia	
(*Annona glabra* L.)		
APOCYNACEAE	Papua	
Carissa papuana Markgr.		
	Indonesia, Papua, Australia	
Cerbera floribunda K. Schum.		
(*Cerbera batjanica* Teijsm. & Binn. ex Valeton)		
	Bangladesh, Southeast Asia, Papua, Australia, Pacific	
Cerbera manghas L.		
(*Cerbera forsteri* Seem., *Cerbera lactaria* Buch.-Ham. ex D. Dietrich		
Cerbera venenifera (Poir.) Steud.,*Elcana seminuda* Blanco,		
Tabernaemontana obtusifolia Poir., *Tanghinia venenifera* Poir.)		
	India, Bangladesh, Southeast Asia, Papua, Australia,	
Cerbera odollam Gaertn.	Pacific	
	Southeast Asia	
Melodinus scandens J.R.Forst. & G.Forst		
(*Melodinus orientalis* Bl.)		
ARALIACEAE		
	Andamans, Southeast Asia	
Schefflera elliptica (Bl.) Harms.		
(*Sciadophyllum ellipticum* Bl.)		
	Malaysia	
Schefflera lanceolata Ridl.		
(*Heptapleurum lanceolatum*(Ridl.) Lowry & G.M. Plunkett)		
	Southeast Asia	
Schefflera ridleyi (King) Viguier		
(*Schefflera musangensis* Hend., *Scheffleropsis polyandra* (Ridley) Ridley)		
ASCLEPIADACEAE		Ringworm (Thailand)[268]
Dischidia bengalensis Colebr.	India, Bangladesh, Southeast Asia, Papua	Cough (Malaysia)[268]
Dischidia major (Vahl) Merr.	India, Bangladesh, Southeast Asia, Australia	
(*Collyris major* Vahl)		Wounds (the Philippines)[268]
Dischidia nummularia R.Br.	India, Bangladesh, Southeast Asia, Australia	
(*Collyris minor* Vahl, ,*Dischidia minor* (Vahl) Merr.,		
Dischidia orbicularis Decne.)		Diarrhoea (Thailand) [265]

Dischidia rafflesiana Wall.	India, Bangladesh, Southeast Asia	
Finlaysonia obovata Wall.<br>(*Finlaysonia maritima* Backer, *Olus crepitans* Rumph.,<br>*Secamone maritima* Bl.)	India, Bangladesh, Southeast Asia	
Gymnanthera nitida R. Br.<br>(*Gymnanthera oblonga* (Burm. f.) P.S. Green)	China, Southeast Asia, Papua, Australia	Burns (Papua)[268]
Hoya australis R. Br. ex J. Traille	Indonesia, Papua, Australia, Pacific	
Hoya coronaria Bl.	Southeast Asia	Fever (Bangladesh)[268]
Hoya parasitica (Roxb.) Wall. ex Wight<br>(*Asclepias parasitica* Roxb.,<br>*Hoya hookeriana* Wight)	India, Bangladesh, Southeast Asia	Gonorrhea (India) [279]
Pentatropis capensis (L. f.) Bullock<br>(*Cynanchum capense* L. f.)	India, Bangladesh, Myanmar	
Sarcolobus carinatus Wall.	India, Bangladesh, Myanmar, Thailand, Malaysia	
Sarcolobus globosus Wall.	Malaysia, Indonesia	
AQUIFOLIACEAE		
Ilex cymosa Bl.	Southeast Asia	
Ilex maingayi Hook.f.	Malaysia	
ASTERACEAE		
Pluchea indica (L.) Less.<br>(*Baccharis indica* L.,<br>*Erigeron denticulatum* Burm. f.)	Tropical Asia and Pacific	Dysentery (Vietnam)[268]
AVICENNIACEAE		
***Avicennia alba* Bl.**<br>(*Avicennia marina* (Forssk.) Vierh., *Avicennia officinalis* L.,<br>*Avicennia tomentosa* Jacq.)	India, Southeast Asia, Papua	Boils (Guinea) [291]
***Avicennia eucalyptifolia* (Valeton) Zipp. ex Moldenke**<br>(*Avicennia officinalis* var. *eucalyptifolia* Valeton)	Papua, Solomons	Boils (Solomons) [281]

***Avicennia lanata* Ridl.**	Malaysia, Indonesia, Papua	
		Boils (Solomons) [281]
***Avicennia officinalis* L.**	India, Bangladesh, Southeast Asia, Papua, Solomons	
**(***Avicennia tomentosa* Jacq.)		
BIGNONIACEAE		Bronchitis (Indonesia)[268]
***Dolichandrone spathacea* (Burm. f.) Bedd.**	India, Bangladesh, Southeast Asia, Papua, Australia,	
(*Bignonia spathacea* L. f.)	Pacific	
BORAGINACEAE		
Cordia cochinchinensis Gagnep.	Vietnam, Cambodia, Thailand	
		Boils (the Philippines)[268]
Cordia dichotoma G. Forst.	India, Bangladesh, Southeast Asia, Papua, Australia	
(Varronia sinensis Lour.)		
		Diarrhoea (India) [269]
CASUARINACEAE	India, Bangladesh, Southeast Asia, Papua, Australia,	
Casuarina equisetifolia L.	Pacific	
(*Casuarina litorea* L. ex Fosberg & Sachet).		
CHENOPODIACEAE		
Atriplex jubata S. Moore	New Caledonia	
Sarcocornia quinqueflora (Bunge ex Ung.-Sternb.)	Australia	
(*Salicornia quinqueflora* Bunge ex Ung.-Sternb.)		
		Infection (Pakistan)[268]
Salicornia brachiata Miq.	India, Bangladesh, Myanmar, Thailand	
		Hepatitis (India)[268]
Suaeda maritima (L.) Dumort.	India, Thailand, Vietnam, Korea. Japan	
(*Chenopodium australe* R. Br., *Chenopodium maritimum* L.)		
CHRYSOBALANACEAE		Diarrhoea (Indonesia) [267]
Atuna racemosa Raf.	Southeast Asia, Papua, Pacific	
COMBRETACEAE	Myanmar, Thailand, Laos, Vietnam	
Combretum quadrangulare Kurz		Trush (the Philippines) [285]
	India, Bangladesh, Southeast, Papua, Australia	
Lumnitzera littoralis (Jack) Voigt		
(*Lumnitzera purpurea* (Gaudich.) C. Presl)		
Lumnitzera racemosa Willd.		
(*Laguncularia purpurea* Gaudich., *Lumnitzera rosea* (Gaudich.) C. Presl)	India, Bangladesh, Southeast, Papua, Australia	

Terminalia catappa L.		Bronchitis (India) [268]
(*Myrobalanus catappa* (L.) Kuntze,		
Terminalia latifolia Blanco, *Terminalia moluccana* Lam.)	India, Bangladesh, Southeast, Papua, Australia	
CONVOLVULACEAE		
		Diarrhoea (Cambodia)[268]
Ipomoea pes-caprae (L.) R. Br.		
(*Convolvulus bilobatus* Roxb., *Convolvulus brasiliensis* L.)		
	India, Bangladesh, Southeast Asia, Papua, Pacific	
CLUSIACEAE		Gonorrhoea (Myanmar)[268]
Calophyllum inophyllum L.		
(*Calophyllum bintagor* Roxb.)		
EBENACEAE	India, Bangladesh, Southeast Asia, Papua, Pacific	
Diospyros areolata King & Gamble		
Diospyros compacta (R. Br.) Kosterm.	Malaysia, Indonesia	
(*Maba compacta* R. Br.)		
Diospyros ferrea (Willd.) Bakh.	Australia	
(*Maba littorea* R. Br., *Maba ebenus* Wight)		
***Diospyros littorea* (R. Br.) Kosterm.**	India, Bangladesh, Southeast Asia, Papua	
		Diarrhoea (India)[268]
(*Maba littorea* R. Br.)		
	Southeast Asia, Papua	
Diospyros malabarica (Descr.) Kostel		
	India, Bangladesh, Southeast Asia	
Diospyros maritima Bl.		
(*Diospyros kusanoi* Hayata,		
Diospyros liukiuensis Makino)		
	Southeast Asia, Taiwan, Papua, Australia	
ERICACEAE		
Rhododendron brookeanum Low		
ex Lindl. var. *brookeanum*		
(*Azalea brookeana* Low ex Lindl.)	Borneo	
EUPHORBIACEAE		
Blumeodendron tokbrai (Bl.) Kurz		
(*Elateriospermum tokbrai* Bl., *Mallotus tokbrai* (Bl.) Müll. Arg.,		
Rottlera tokbrai (Bl.) Scheff.)	Indonesia, Papua	

Breynia racemosa (Bl.) Muell. Arg.	Southeast Asia, Papua	Leprosy (Papua) [291]
Croton heterocarpus Müll. Arg.<br>(*Oxydectes heterocarpa* (Müll. Arg.) Kuntze	Malaysia, Indonesia	
Excoecaria agallocha* L.**<br>**(Commia cochinchinensis* Lour.)	India, Bangladesh, Southeast Asia, Papua, Australia, Pacific	
***Excoecaria indica* (Willd.) Müll. Arg.**<br>(*Sapium indicum* Willd.)	India, Bangladesh, Southeast Asia, Papua	
Suregada glomerulata (Bl.) Baill.	Southeast Asia, Papua, Australia	Boils (Bangladesh)[268]
FABACEAE		
Caesalpinia bonduc (L.) Roxb.<br>(*Caesalpinia bonducella* (L.) Fleming,<br>*Guilandina bonduc* L.)	India, Bangladesh, Southeast Asia, Papua, Australia, Pacific	
Canavalia cathartica Thouars<br>(*Lablab microcarpus* DC.)	India, Bangladesh, Southeast Asia, Papua, Australia, Pacific	
Canavalia lineata (Thunb.) DC.<br>(*Dolichos lineatus* Thunb.	Bangladesh, Myanmar, Thailand, Vietnam, China, Korea, Japan	
Canavalia maritima Thouars<br>(*Canavalia obtusifolia* (Lam.) DC.)	India, Bangladesh, Southeast Asia, Papua, Australia, Pacific	
Cathormion umbellatum (Vahl) Kosterm.<br>(*Albizia umbellata* (Vahl) E. J. M. Koenen)	India, Bangladesh, Southeast Asia, Papua, Australia	
***Cynometra iripa* Kostel**.	India, Bangladesh, Southeast Asia, Papua, Australia	
Cynometra ramiflora Miq.	India, Bangladesh, Southeast Asia, Papua, Australia	
Dalbergia candenatensis (Dennst.) Prain<br>(*Cassia candenatensis* Dennst.)	India, Bangladesh, Southeast Asia, Papua, Australia, Pacific	Fever (Indonesia)[268]
Derris heptaphylla Merr.	India, Bangladesh, Southeast Asia, Papua	
Derris pinnata (Lour.) Prain<br>(*Dalbergia pinnata* (Lour.) Prain)	India, Bangladesh, Southeast Asia, China	
Derris scandens (Aubl.) Pittier<br>(*Deguelia scandens* Aubl.)	India, Bangladesh, Southeast Asia, Papua, Australia	

Derris trifoliata Lour.<br>(*Pongamia uliginosa* (Roxb. ex Willd.) DC.,<br>*Dalbergia heterophylla* Willd.,<br>*Deguelia uliginosa* (Roxb. ex Willd.) Baill.)	India, Bangladesh, Southeast Asia, Papua	UTI (Solomons) [286]
Inocarpus fagifer (Parkinson) Fosb<br>(*Inocarpus edulis* J.R. Forst. & G. Forst)	Southeast Asia, Papua, Pacific	Diarrhoea (Fiji) [270]
Intsia bijuga (Colebr.) Kuntze<br>(*Macrolobium bijugum* Colebr.)	India, Bangladesh, Southeast Asia, Papua, Australia, Pacific	Diarrhoea (India) [269]
Pongamia pinnata (L.) Pierre<br>(*Cytisus pinnatus* L., *Derris indica* (Lam.) Bennet,<br>*Milletia pinnata* (Linnaeus) Panigrahi, *Pongamia glabra* Vent.)	India, Bangladesh, Southeast Asia, Papua, Australia, Pacific	
Serianthes grandiflora Benth.<br>(*Serianthes dilmyi* Fosberg)	Southeast Asia, Papua	
Sindora siamensis Teysm. ex Miq.	India, Bangladesh, Myanmar, Thailand, Cambodia, Vietnam	
Wallaceodendron celebicum Koord.	Indonesia, the Philippines	
FLACOURTIACEAE<br>*Hydnocarpus ilicifolia* King	Thailand, Vietnam, Malaysia	
Scolopia luzonensis (Presl.) Warb.	Indonesia, the Philippines	
Scolopia macrophylla (W. & A.) Clos	Myanmar, Thailand, Cambodia, Vietnam, Malaysia, Indonesia	
Xylosma suluense Merr.	Malaysia, Indonesia, the Philippines	Fever (Samoa) [268]
GENTIANACEAE<br>*Fagraea crenulata* Maingay ex C.B. Clarke	Vietnam, Thailand, Malaysia, Indonesia	
Fagraea berteroana A. Gray ex Benth.	Pacific	
GOODENIACEAE<br>*Scaevola hainanensis* Hance	Vietnam, China	Dysentery (the Philippines)[268]
Scaevola taccada (Gaertn.) Roxb.	India, Bangladesh, Southeast Asia, Papua, Australia, Pacific	

(*Lobelia frutescens* Mill., *Lobelia taccada* Gaertn., *Scaevola piliplena* Miq.,		
Scaevola sericea Vahl)		
HERNANDIACEAE		UTI (Fiji)[268]
	Southeast Asia, Papua, Australia, Pacific	
Hernandia ovigera L.		
(*Hernandia sonora* L.)		
	Tropical Asia and Pacific	
Hernandia nymphaeifolia (C. Presl) Kubitzki		
(*Biasolettia nymphaeifolia* C. Presl,		
Hernandia peltata Meisn.)		
ICACINACEAE	Indonesia, Philippines, Papua	
Merrilliodendron megacarpum Sleumer		Syphilis (Vietnam) [287]
(*Stemonurus megacarpus* Hemsl.)		
LAURACEAE	India, Bangladesh, Southeast Asia, Papua, Australia,	
Cassytha filiformis L.	Pacific	
(*Calodium cochinchinense* Lour., *Cassytha zeylanica* Gaertn.,		Cholera (Bangladesh)[268]
Cassytha dissitiflora Meisn, *Cassytha aphylla* (Forssk.) Raeusch.)		
LECYTHIDACEAE		
	India, Bangladesh, Southeast Asia, Papua, Australia,	
Barringtonia acutangula (L.) Gaertn.	Pacific	
(*Barringtonia luzonensis* (Presl) Vidal)		
	India, Bangladesh, Southeast Asia, Papua, Australia,	
Barringtonia asiatica (L.) Kurz	Pacific	
(*Barringtonia butonica* J.R. Forst.,		
Barringtonia speciosa J.R. Forst. & G. Forst.)		
	Myanmar, Malaysia, Indonesia	
Barringtonia conoidea Griff.		Otitis (Palau)[268]
(*Barringtonia alata* Wall.)		
	Southeast Asia, Papua	
Barringtonia macrostachya Hook.f.		
	India to Pacific	
Barringtonia racemosa (L.) Spreng		
(*Barringtonia ceylanica* (Miers) Gardner ex C.B. Clarke,		
Barringtonia elongata Korth., *Barringtonia timorensis* Bl.)		
LOPHOPYXIDACEAE	Southeast Asia, Papua	
Lophopyxis maingayi Hook.f.		
	Indonesia	
LORANTHACEAE		

***Amyema anisomeres* Danser**	Southeast Asia	
		Wounds (Malaysia)[268]
***Amyema gravis* Danser**	Indonesia, Papua, Australia	
(*Loranthus obovatus* Schröt. & Back.)		
***Amyema mackayensis* (Blakely) Danser**	Bangladesh, Southeast Asia, China	
(*Loranthus mackayensis* Blakely)		
Dendrophthoe pentandra (L.) Miq.	China, Southeast Asia, Papua	
(*Loranthus pentandrus* L.)		
Macrosolen cochinchinensis (Lour.) Tiegh.	Bangladesh, Southeast Asia, China, Australia	
(*Loranthus cochinchinensis* Lour.)		
Viscum ovalifolium DC	India	
(*Viscum pedunculatum* Barlow)		
Viscum orientale Willd.	India, Bangladesh, Southeast Asia, Papua, Australia	
LYTHRACEAE		
***Pemphis acidula* J.R. & G. Forst.**		Diarrhoea (Bangladesh) [271]
(*Lythrum pemphis* L.f., *Pemphis angustifolia* Roxb.,		
Pemphis setosa Blanco)	Southeast Asia, Papua, Pacific	
		Leprosy (New Guinea) [291]
MALVACEAE	India, Bangladesh, Southeast Asia	
***Brownlowia argentata* Kurz**		
	Papua, Australia	
***Brownlowia tersa* (L.) Kosterm**		Dysentery (the Philippines)[268]
(*Glabraria tersa* L.)	Indonesia, the Philippines, Papua	
***Camptostemon schultzii* Mast.**		
	Bangladesh, Southeast Asia, China, Pacific	*Eye infection (Solomons) [289]*
***Camptostemon philippinense* (S. Vidal) Becc.**		
		Diarrhoea (India) [272]
(*Cumingia philippinensis* S. Vidal)		
	Bangladesh, Vietnam, China, Malaysia	Colds (Solomons)[268]
Hibiscus tiliaceus L.		
(*Hibiscus boninensis* Nakai, *Hibiscus tortuosus* Roxb.,	India, Bangladesh, Myanmar, Thailand, Malaysia	
Talipariti tiliaceum (L.) Fryxell)		
Kleinhovia hospita L.	India, Bangladesh, Southeast Asia, Papua, Australia, Pacific	

Malachra capitata (L.) L.		Post-partum (Vietnam) [272]
(Sida capitata L.)		
Thespesia populnea (L.) Soland. ex Correa		
(*Hibiscus baccifer* Bl., *Hibiscus populneus* L.,	Myanmar, Thailand, Cambodia, Vietnam	
Thespesia macrophylla Bl.,*Thespesia populneoides* (Roxb.) Kostel.)		
	Vietnam, Malaysia, Borneo	
MELASTOMATACEAE		
	Southeast Asia	
Melastoma saigonense (Kuntze) Merr.		Dysentery (India)[268]
(*Osbeckia saigonensis* Kuntze)		
Ochthocharis borneensis Bl.		
	India, Bangladesh, Southeast Asia, Papua	Dysentery (Yap)[268]
Pachycentria constricta (Bl.) Bl.		
(*Melastoma constrictum* Bl.)		
MELIACEAE	India, Bangladesh, Southeast Asia, Papua, Pacific	Cholera (Myanmar)[268]
***Aglaia cucullata* Pellegr.**		
(Amoora cucullata Roxb.,*Aphanamixis cucullata* (Roxb.) M.R. Almeida)		
***Xylocarpus granatum* J. Koenig**	India, Bangladesh, Southeast Asia, Papua, Australia,	
(*Carapa granatum* (J. Koenig) Alston,	Pacific	
Carapa obovata Bl.,*Xylocarpus obovatus* Juss)		
***Xylocarpus moluccensis* (Lam.) M. Roem.**	India, Bangladesh, Southeast Asia, Papua, Australia,	
(*Carapa moluccensis* Lam.,	Pacific	
Xylocarpus mekongensis Pierre)		Diarrhoea (China)[269]
	India, Bangladesh, Southeast Asia, Papua, Australia,	
***Xylocarpus rumphii* (Kostel.) Mabb.**	Pacific	
	Southeast Asia, China, Papua, Australia, Pacific	
MORACEAE		
Ficus microcarpa L.f.		
(*Ficus littoralis* Bl., *Ficus retusa* L., *Ficus nitida* Thunb.)		
Malaisia scandens (Lour.) Planch	Vietnam, China, Japan	
(*Caturus scandens* Lour., *Malaisia tortuosa* Blanco,		
Trophis scandens (Lour.) Hook. & Arn.)		
	New Caledonia	
MYOPORACEAE		
Myoporum bontioides (Siebold & Zucc.) A. Gray		
	Sri Lanka, Southeast Asia, Papua	
(*Polycoelium bontioides* (Siebold & Zucc.) A. DC.)		

	Papua	
Myoporum tenuifolium G.Forsl.		
MYRISTICACEAE<br>*Horsfieldia irya* (Gaertn.) Warb.	Myanmar, Thailand, Cambodia, Vietnam	Wounds (Indonesia)[268]
Myristica hollrungii Warb.		
	Southeast Asia, Papua, Australia	
MYRSINACEAE		
Rapanea porteriana (Wall. ex A. DC.) Mez<br>(*Myrsine porteriana* Wall. ex A. DC.)	Southeast Asia, Papua, Australia, Pacific	
MYRTACEAE<br>*Melaleuca cajuputi* Roxb.	Indonesia, the Philippines, Papua, Australia	
Melaleuca quinquenervia (Cav.) S.T. Blake<br>(*Metrosideros quinquenervia* Cav.)	Southeast Asia	
	India, Sri Lanka, Bangladesh, Southeast Asia, Papua, Pacific	
***Osbornia octodonta* F. Muell.**		
OLACAEAE		
Anacolosa frutescens (Bl.) Bl.<br>(*Stemonurus frutescens* Bl.)	India, Sri Lanka, Bangladesh, Southeast Asia	Fever (China)[268]
Olax imbricata Roxb.<br>(*Olax wightiana* Wall. ex Wight & Arn.,<br>*Ximenia olacoides* Wight & Arn.)	India, Sri Lanka, Bangladesh, Southeast Asia<br><br>India, Sri Lanka, Bangladesh, Southeast Asia, Papua, Pacific	
Olax scandens Roxb.		Tonsilitis (Indonesia)[268]
Strombosia ceylanica Gardner		
Ximenia americana L.<br>(*Ximenia aculeata* Crantz, *Ximenia elliptica* G. Forst.)	India, Sri Lanka, Bangladesh, Southeast Asia	
PHYLLANTHACEAE		
Glochidion littorale Bl.<br>(*Bradleia littorea* (Bl.) Steud.,<br>*Diasperus litoralis* (Bl.) Kuntze,<br>*Phyllanthus littoralis* (Bl.) Müll. Arg.)	Indonesia, Papua, Australia<br><br>India, Bangladesh, Myanmar, Thailand, Malaysia	
		Cough (Thailand)[268]
PLUMBAGINACEAE<br>***Aegialitis annulata* R.Br.**	Korea, Japan	

(*Aegialinites annulata* (R. Br.) C. Presl)		
***Aegialitis rotundifolia* Roxb.**	India, Bangladesh, Southeast Asia, Papua, Australia	
Limonium tetragonum (Thunb.) Bullock		Dysentery (Palau)[268]
RHIZOPHORACEAE	Indonesia, Papua, Australia	
***Bruguiera cylindrica* (L.) Bl.**		
(*Bruguiera caryophylloides* (Burm. f.) Bl., *Rhizophora cylindrica* L., *Rhizophora cylindrica* L.)	India, Bangladesh, Southeast Asia, Papua, Australia, Pacific	
		Burns (India)[268]
***Bruguiera exaristata* Ding Hou**	Southeast Asia, Papua, Australia	
	Malaysia	
***Bruguiera gymnorhiza* (L.) Savigny**		Wounds (India)[268]
(*Rhizophora gymnorhiza* L.)	India, Bangladesh, Southeast Asia, Papua, Australia	
		Fever (Malaysia)[268]
***Bruguiera hainesii* C.G.Rogers**		
***Bruguiera paviflora* (Roxb.) Wight.**	India, Bangladesh, Southeast Asia, Papua, Australia	Sores (Australia) [280]
***Bruguiera sexangula* (Lour.) Poir.**	Indonesia, Papua, Australia	
(*Bruguiera eriopetala* Wight & Arn., *Bruguiera sexangula* var. *rhynchopetala* W.C. Ko,*Rhizophora sexangula* Lour.)		
Carallia brachiata (Lour.) Merr.	Indonesia, Papua, Australia	Postpartum (Malaysia)[268]
Carallia suffruticosa Ridl.		
(*Carallia fascicularis* Guill)		
	Malaysia	
***Ceriops australis* (C.T. White)**		
**Ballment, T.J.Sm. & J.A. Stoddart**	India, Southeast Asia, Papua, Australia	
(*Ceriops tagal* var. *australis* C.T. White)		
		Postpartum (Malaysia)[268]
***Ceriops decandra* (Griff.) Ding Hou**	India, Southeast Asia, Papua	
***Ceriops tagal* (Perr.) C.B. Rob.**	Vietnam, China, Japan	
(*Ceriops candolleana* Arn.)		
	Southeast Asia, China, Papua, Australia	
Kandelia candel (L.) Druce		
(*Kandelia rheedi* Wight & Arn., *Rhizophora candel* L.)		

Kandelia obovata Sheue H.Y. Liu & J. Yong	Southeast Asia, China, Papua, Australia	
***Rhizophora apiculata* Bl.**<br>(*Rhizophora candelaria* DC.,*Rhizophora conjugata* L., *Rhizophora mangle* L., *Rhizophora mucronata* Lam., *Rhizophora longissima* Blanco, *Rhizophora macrorrhiza* Griff.)	India, Bangladesh, Myanmar	Abscess (Cambodia)[268]
***Rhizophora stylosa* Griff.**<br>(*Rhizophora mucronata* var. *stylosa* (Griff.) Schimp.)	India, Bangladesh, Southeast Asia, Papua	
RUBIACEAE		
Gardenia lucida Roxb.<br>(*Gardenia resinifera* Roth)	Southeast Asia, Papua	Chancre (Palau)[268]
Guettarda speciosa L.<br>(*Matthiola speciosa* (L.) Kuntze)	Indonesia, Papua, Australia	
Hydnophytum formicarum Jack<br>(*Hydnophytum orbiculatum* Elmer, *Lasiostoma formicarum* (Jack) Spreng.)	India, Bangladesh, Southeast Asia, Papua, Australia, Pacific	
Ixora timorensis Decne.	Southeast Asia, Papua, Pacific	
Morinda citrifolia L.<br>(*Morinda bracteata* Roxb., *Morinda ligulata* Blanco, *Morinda multiflora* Roxb.)	Thailand, Cambodia, Vietnam, China, Japan	
Myrmecodia tuberosa Jack<br>(*Lasiostoma tuberosum* (Jack) Spreng.)	India, Bangladesh, Southeast Asia, Papua, Australia, Pacific	Dysentery (India) [270]
Psychotria serpens L.<br>(*Psychotria scandens* Hook. & Arn.)	India, Bangladesh, Southeast Asia, China, Papua, Australia	
Scyphiphora hydrophyllacea C.F. Gaertn.		
RUTACEAE	India, Bangladesh, Myanmar, Thailand, Cambodia, Vietnam, Malaysia	
Acronychia pedunculata (L.) Miq.<br>(*Acronychia laurifolia* Bl., *Acronychia apiculata* Miq., *Jambolifera pedunculata* L.)	Vietnam	
Atalantia monophylla DC.<br>(*Limonia monophylla* L.)	India, Bangladesh, Southeast Asia, Papua	
Limnocitrus littoralis (Miq.) Swingle		Fever (Thailand)[268]

(*Pleiospermium littorale* (Miq.) Tanaka)	India, Cambodia , Southeast Asia	
Merope angulata Swingle		
	India, Bangladesh, Myanmar, Thailand	
SALVADORACEAE<br>*Azima sarmentosa* (Bl.) B. & H.<br>(*Actegeton sarmentosa* Bl.)	India, Southeast Asia	
Azima tetracantha Lam.		
	India, Bangladesh, Southeast Asia, China, Papua, Australia, Pacific	
SAPINDACEAE<br>*Allophylus cobbe* (L.) Raeusch<br>(*Rhus cobbe* L.)	Papua	
Harpullia arborea (Blanco) Radlk<br>(*Ptelea arborea* Blanco)	Papua	
	Papua	
Harpullia crustacea Radlk.		
	Southeast Asia, Papua	
Harpullia hirsuta Radlk.		
Harpullia leptococca Radlk.	Bangladesh, Nicobar Islands, Southeast Asia, Papua New, Guinea, Australia	
Mischocarpus sundaicus Bl.		
	Cambodia, Vietnam	
Planchonella obovata (R.Br.) Pierre.<br>(*Sersalisia obovata* R. Br.)		
	Bangladesh, Southeast Asia, Papua	
SIMAROUBACEAE<br>*Quassia harmandiana* (Pierre) Nooteboom<br>(*Samadera harmandiana* Pierre)		Leprosy (India)[268]
	Pacific	
Quassia indica (Gaertn.) Nooteboom<br>(*Samadera indica* Gaertn., *Vittmania elliptica* Vahl)	Pacific	
SOLANACEAE<br>*Solanum inamoenum* Benth.	India, Sri Lanka, Bangladesh, Myanmar	
Solanum viride R.Br.	Southeast Asia, Papua, Australia, Pacific Islands	
SONNERATIACEAE<br>***Sonneratia apetala* Buch.-Ham**.		
***Sonneratia griffithii* Kurtz.**		

(*Chiratia leucantha* Montrouz., *Sonneratia acida* Benth., *Sonneratia alba* J. Smith,*Sonneratia caseolaris* (L.) Engl., *Sonneratia iriomotensis* Masam.,*Sonneratia mossambicensis* Klotzsch ex Peters, *Sonneratia pagatpat* Blanco)<br><br>***Sonneratia ovata* Back.**<br><br>STERCULIACEAE<br>***Heritera littoralis* Aiton**<br>(*Balanopteris minor* Gaertn.,<br>*Balanopteris totila* Gaertn., *Heriteria minor* Roxb.<br><br>***Heritiera fomes* Buch. Ham.**<br><br>***Heritiera globosa* Kostermans**<br><br>STYRACACEAE<br>*Styrax agrestis* (Lour.) G. Don<br>(*Cyrta agrestis* Lour.,*Styrax rostratus* Hosok.,*Styrax subcrenatus* Hand.-Mazz.)<br><br>SYMPLOCACEAE<br>*Symplocos celastrifolia* Griff. ex Clarke<br><br><br>STERMONURACEAE<br>*Stemonurus ammui* Sleumer<br><br>VERBENACEAE<br>*Premna odorata* Blanco<br>(*Gumira odorata* (Blanco) Kuntze,*Premna vestita* Schauer)<br><br>*Premna integrifolia* L.<br>(*Premna corymbosa* Rottler & Willd., *Premna serratifolia* L.)<br><br>VITACEAE<br>*Cayratia trifolia* (L.) Domin<br>(*Causonis trifolia* (L.) Mabb. & J.Wen, *Vitis trifolia* L.)	Southeast Asia, China<br><br>India, Sri Lanka, Bangladesh, Southeast Asia, Papua<br><br>India, Bangladesh, Myanmar, Myanmar, Malaysia<br><br>Borneo<br><br>Vietnam<br><br>India, Bangladesh, Southeast Asia, China, Papua, Australia, Pacific<br><br>Papua<br><br>India, Bangladesh, Southeast Asia, China, Papua, Australia<br><br>India, Bangladesh, Southeast Asia, China, Papua, Australia, Pacific<br><br>India, Bangladesh, Southeast Asia, China, Papua, Australia, Pacific	Diarrhoea (Bangladesh)[268]<br><br>Tuberculosis (the Philippines)[268]<br><br>Fever (Papua)[268]<br><br>Abscess (Thailand)[268]

Bold: true mangrove plants [3]
