# Supplementary material for: Antimicrobial Secondary Metabolites from the Mangrove Plants of Asia and the Pacific
Source: Mar Drugs. 2022 Oct 15;20(10):643. doi: 10.3390/md20100643 (PMC9605323; doi:10.3390/md20100643)
Supplement: Supplementary file 1 [file marinedrugs-20-00643-s001.zip › Table S2.pdf]

Table S2. Antimicrobial activity of extracts and isolates from the Dicotyledons from the mangroves, tidal rivers, and the seashores of Asia and the Pacific

FAMILY<br>Genus, species<br>(synonym)	Extract			Antimicrobial principle(s)
	Antibacterial	Antifungal	Antiviral	
ACANTHACEAE<br>***Acanthus ebracteatus* Vahl**	●	●	●	*Antibacterial:* 3,5-Dimethoxy-4-hydroxy methyl benzoic acid (**74**), (*Z*)-4-coumaric acid 4-*O*-β-*D*-glucopyranoside (**75**), 6-hydroxybenzoxazolinone (**76**) [241].
AEGICERATACEAE<br>***Aegiceras corniculatum* (L.) Blanco**	●			*Antibacterial:* Acornine 2 (**63**) *C. albicans*, *T. mentagrophytes*, *T. rubrum*, *S. cerevisae*, *T. clypeatus* at 50, 50, 100, 9.3, and 150 µg/disc, respectively [212].
AIZOACEAE<br>*Sesuvium portulacastrum* (L.) L.	●	●	●	
ANNONACEAE<br>*Annona palustris* L.	●	●		*Antimicrobial:* Kauranes [26]
APOCYNACEAE<br>*Cerbera manghas* L.	●	●		
Cerbera odollam Gaertn.	●	●		
ASCLEPIADACEAE<br>*Hoya parasitica* (Roxb.) Wall. ex Wight	●			
ASTERACEAE<br>*Pluchea indica* (L.) Less.	●			
AVICENNIACEAE<br>***Avicennia alba* Bl.**	●	●		
***Avicennia eucalyptifolia* (Valeton) Zipp. ex Moldenke**	●			
***Avicennia lanata* Ridl.**	●			
***Avicennia officinalis* L.**	●			*Antibacterial:* Rhizophorin B, *B. subtilis* at 500 µg/mL [246].<br>*Antifungal:* Excoecarin A (**77**), *ent*-16-hydrozy-3-oxo-13-*epi*-manoyl-oxide (**78**), *ent*-15-hydroxy-labda-8(17), 13*E*-dien-3-one (**79**), *R. orizae* and *A. niger* at 500 µg/mL [246].
BIGNONIACEAE<br>***Dolichandrone spathacea* (Burm. f.) Bedd.**	●	●		*Antibacterial:* Decaffeoyl acteoside (**80**) and verbascoside (**81**), *E. faecalis* and *S. sonnei* MIC: 31.2 µg/mL [247].<br>*Antiviral*: Verbascoside (**81**), RSV, EC_{50}: 0.8 µg/mL [248].

BORAGINACEAE				
Cordia dichotoma G. Forst.	•	•		
CASUARINACEAE				
Casuarina equisetifolia L.	•	•		
CHENOPODIACEAE				
Salicornia brachiata Miq.	•	•	•	
Suaeda maritima (L.) Dumort.	•	•		
COMBRETACEAE				
Combretum quadrangulare Kurz	•	•	•	*Antibacterial:* Corilagin (**38**), *S. aureus*, IZD: 12 mm (100 µg/6 mm disc) [81], *S. aureus*, *B. subtilis*, MIC : 50 and 400 µg/mL, respectively [126]. *Antifungal:* Corilagin (**38**), *C. albicans*, IZD: 12 mm (100 µg/6 mm disc) [81]. *Antiviral:* Corilagin (**38**), CV A16 and EV-71, IC_{50} : 32.3 and 5.6 µg/mL, respectively [127]. Corilagin (**38**), IAV, IC_{50}: 31.2 µg/mL [128].
Lumnitzera racemosa Willd.	•			
Terminalia catappa L.	•	•	•	
CONVOLVULACEAE				
Ipomoea pes-caprae (L.) R. Br.	•	•		
CLUSIACEAE				
Calophyllum inophyllum L.				*Antibacterial:* Caloxanthone A, calophynic acid, brasiliensic acid, I nophylloidic acid, calaustralin, calophyllolide, inophyllum C, and inophyllum E (20 µg/disc) *S. aureus*, IZD: 9, 10, 11, 9, 11, 16, 10, and 13 mm, respectively [60]. *Antiviral:* Inophyllum B (**15**), inophyllum B acetate (**16**), and inophyllum P (**17**), reverse transcriptase IC_{50}: 0.03, 0.7, and 0.1 µM, respectively. Inophyllum B and P, HIV-1, IC_{50}: 1.4 and 1.6 µM, respectively [61].
EBENACEAE				
***Diospyros littorea* (R. Br.) Kosterm.**	•			
EUPHORBIACEAE				
***Excoecaria agallocha* L.**	•	•	•	*Antiviral:* 12-Deoxyphorbol 13-(3*E*,5*E*-decadienoate) (**18**), HIV-1, IC_{50}: 6 nM [69]. *Antiviral:* 5β-carboxymethyl-3α-hydroxy-2β-hydroxymethyl-1-methylpyrrolidine (**19**), HIV-1, 10^{-5} M by 22.4% [70].

Suregada glomerulata (Bl.) Baill.				
FABACEAE<br>*Caesalpinia bonduc* (L.) Roxb.	•	•	•	*Antibacterial:* Bondenolide (**23**) (1 mg/mL/agar well), *P. aeruginosa* 7 mm. Neocaesalpin P (**24**), *S. aureus*, *S. agalactiae*, and *P.aeruginosa* MIC: 16, 16, and 32 *µg/mL, respectively [96].*<br>*Antifungal:* Bondenolide (**23**) (200 µg/mL) *N. oryzae, S. atra, C. lunata, D. rostrata*, *A. niger*, *P. oustreatus*, *A. boydii*, *M. canis*, and *E. flocossum* [96].
	•			
Canavalia cathartica Thouars	•			
Cathormion umbellatum (Vahl) Kosterm.	•	•	•	
***Cynometra iripa* Kostel**.	•		•	
Cynometra ramiflora Miq.				
Dalbergia candenatensis (Dennst.) Prain				*Antibacterial and antifungal:* Mucronatol, *E. coli*, *T. mentagrophytes* MIC: 50 and 25µg/mL, respectively. Clausequinone *S. aureus*, *B. subtili* , *E. coli*, *A. niger*, and *T. mentagrophytes* MIC: 6, 3, 6, 50, 100, and 25 µg/mL, respectively. Vestitol, *B. subtilis*, *E. coli*, and *T. mentagrophytes* MIC: 50, 50 and 25 µg/mL, respectively [106].
	•	•	•	
Derris scandens (Aubl.) Pittier				*Antibacterial:* Lupalbigenin (**25**), *S. aureus* and MRSA, MIC: 2 and 4 µg/mL, respectively. Derrisisoflavone A (**26**) , *S. aureus* and MRSA, MIC: 16 and 4 µg/mL, respectively. Santal (**27**), MRSA, MIC: 2 µg/mL. Scandenin A (**28**) (50 µL of 1 mg/mL/disc) *E. coli,* 9 mm [108]. Dalpanitin (**29**) and vicenin 3 (**30**), *S. aureus*, MIC: 23 µg/mL [109,110]<br>*Antifungal:* Derrisisoflavone C (**31**), 5,7,4′-trihydroxy-6,8-diprenylisoflavone (**32**) and lupalbigenin (**25**), 250 µg/mL *T. mentagrophytes* [107].
	•	•		
Derris trifoliata Lour.				*Antibacterial:* Lupinifolin (**35**), *S. aureus*, *B. subtilis*, *E. coli*, and *P. aeruginosa* 0.5, 0.5, 10, and 0.5 µg/TLC. *S. aureus*, MIC: 8 µg/mL [115,116].<br>*Antifungal:* Lupinifolin (**35**), *C. albicans*, 0.01 µg/TLC [116].<br>*Antiviral:* Rotenone (**34**) HSV-1 and HSV-2; 15.4 and 24.4 % at 50 µg/mL, respectively [117]. Deguelin (**33**), HCMV; IC_{50}: 55.8 nM [117].
	•			
Inocarpus fagifer (Parkinson) Fosb	•	•	•	
Pongamia pinnata (L.) Pierre	•			

Sindora siamensis Teysm. ex Miq.	●	●	●	
GOODENIACEAE				
Scaevola taccada (Gaertn.) Roxb.				*Antifungal:* Scataccanol (**87**), *P. insidiosum*, MIC: 5 μg/mL. 4-Formylsyringol (**88**), *P. insidiosum* MIC: 10 μg/mL [261].
HERNANDIACEAE				
Hernandia nymphaeifolia (C. Presl) Kubitzki				*Antiviral:* Deoxypodophyllotoxin (**3**), HSV-1, IC_{50}: 0.004 μM [25].
ICACINACEAE				
Merrilliodendron megacarpum Sleumer				*Antifungal:* Camptothecin (**66**), *A. alternata*, *E. nigrum*, *P. guepinii*, *Drechslera sp.*, and *Fusarium avenaceum*, EC_{50} <25, <10, 10, <10, and 30 μg/mL, respectively [214].<br>*Antiviral:* Camptothecin (**66**), VV, 40 μM [215], EV 71, 10 μM [216]. 10 μg/mL, IVA [217], SIM [218], HIV [219].
LAURACEAE				
Cassytha filiformis L.	●	●		*Antifungal: Dicentrine (***2***),C. clodosporioides*, 6 μg/spot [24].
LECYTHIDACEAE	●	●		
Barringtonia acutangula (L.) Gaertn.				
Barringtonia asiatica (L.) Kurz				*Antibacterial:* Germanicol caffeoyl ester (**61**), camelliagenone (**62**), germanicol (**63**), *P. aeruginosa* [207]<br>*Antifungal:* Germanicol caffeoyl ester (**61**), camelliagenone (**62**), germanicol (**63**), *C. albicans* [207].
	●		●	
Barringtonia racemosa (L.) Spreng				*Antibacterial:* Nasimalun A (**64**) (200 μg/disc) *S. aureus*, *B. subtilis*, *S. flexneri*, *S. paratyphi* B, *V. mimicus*, IZD: 9, 10, 7, 10, and 11 mm, respectively [204]. Lupeol (**40**), *E. faecalis*, MIC_{50} of 62.5 μg/mL [208].
LORANTHACEAE				
Dendrophthoe pentandra (L.) Miq.	●			*Antibacterial:*Flavonol glycosides [186].
	●			
Macrosolen cochinchinensis (Lour.) Tiegh.				
Viscum orientale Willd.	●			
LYTHRACEAE				

***Pemphis acidula* J.R. & G. Forst.**	●	●		
MALVACEAE	●			
Hibiscus tiliaceus L.				
Kleinhovia hospita L.			●	*Antiviral:* (9*R*,10*R*, 23*R*)-21,23:23,27-diepoxycycloarta-1,24-diene-3,27-dione (**49**) and (9*R*,10*R*,21*S*,23*R*)-21/23,23/27-diepoxy-21-methoxycycloartan-1,24-diene-3,27-dione (**50**), HIV, IC_{50}: 0.8 and 2.4 μM [156].
Malachra capitata (L.) L.	●	●	●	
Thespesia populnea (L.) Soland. ex Correa	●			*Antibacterial:* Populene C (**42**), populene D (**43**), mansonone D (**44**), mansonone E (**45**), 7-hydroxycadalene (**46**), gossypol (**47**), and (+) 6,6′-methoxygossypol (**48**), *B. cereus* MIC: 4.6, 4.6, 2.3, 4.6, 0.5, 1.1, and 2.3 μg/mL, respectively. Gossypol (**47**) and (+) 6,6′-methoxygossypol (**48**), *S. aureus*, MIC: 1.1 and 4.6 μg/mL, respectively. (+) 6,6′-Methoxygossypol (**48**), *E. faecalis* MIC: 1.1 μg/mL [152].
MELIACEAE	●	●	●	
***Aglaia cucullata* Pellegr.**				
***Xylocarpus granatum* J. Koenig**				*Antifungal:* 3-(1-Hydroxyethyl)-2,2-dimethyl-4-butyrolactone (**59**), 20 μg/mL, *B. graminis* by 67.4% [171].<br>*Antiviral:* Sundarbanxylogranin B (**55**), HIV-1, IC_{50}: 23.1 μM [167].
***Xylocarpus moluccensis* (Lam.) M. Roem.**				*Antiviral:* Thaixylomolin I (**56**), K (**57**), IVA, IC_{50}: 77.1, 113.5 μM [168]. Krishnolide A (**58**), 20 μM, HIV-1, 79.7% [169].
MORACEAE				
Ficus microcarpa L.f.				*Antiviral:* (+)(2*R*,3*S*) afzelechin (**36**) and (-)(2*R*,3*R*) epiafzelechin (**37**), HSV-1, IC_{50}: 490 and 550 μg/mL [122].
MYOPORACEAE				
Myoporum bontioides (Siebold & Zucc.) A. Gray	●	●		*Antibacterial:* Myoporumine A (**82**), myoporumine B (**83**), (−)-epingaione (**84**), and (−)-dehydroepingaione (**85**), MRSA, MIC: 6.2, 6.2, 25, and 25 μg/mL, respectively [250].<br>*Antifungal:* (−)-Epingaione (**84**), *F. oxysporum*, *Pestalotia mangiferae* (EC_{50}: 77 mg/L), *T. paradoxa* , *C.musae* , *A. alternata* , *M. sentina*, and *S. fawcettii* [249]. homomonoterpene, *P. capsici*, IC_{50} below 63.5 μg/mL [251].
MYRTACEAE	●	●		
Melaleuca cajuputi Roxb.				
Melaleuca quinquenervia (Cav.) S.T. Blake	●	●	●	
	●			

OLACAEAE				
Olax scandens Roxb.	•	•		
Ximenia americana L.	•	•		
PLUMBAGINACEAE				
***Aegialitis rotundifolia* Roxb.**	•		•	
Limonium tetragonum (Thunb.) Bullock	•		•	
RHIZOPHORACEAE				
***Bruguiera cylindrica* (L.) Bl.**	•<br>•		•	
***Bruguiera gymnorhiza* (L.) Savigny**			•	
***Bruguiera sexangula* (Lour.) Poir.**	•			
Carallia brachiata (Lour.) Merr.	•			
***Ceriops decandra* (Griff.) Ding Hou**		•	•	*Antibacterial:* 16-Hydroxypimar-8(14)-en-15-one (**22**), *B. cereus*, *S.* aureus, *M. kristinae*, *S. pyogenes*, and *S. pooni*, MIC:100, 100, 100, 500, and 250 μg/mL, respectively [80].
***Ceriops tagal* (Perr.) C.B. Rob.**				
Kandelia candel (L.) Druce				
***Rhizophora apiculata* Bl.**	•			*Antibacterial:* 2,6-Dimethoxy-*p*-benzoquinone (**20**) [78]. Gallic acid (**21**), *K.* pneumoniae, *mirabilis, Proteus vulgaris, S. marcesens*, MIC 250, 500, 125, and 250 μg/mL, respectively [86]; *S. aureus*, MIC: 64 μg/mL, respectively, MRSA, MIC: 128 μg/mL [87]. *S. aureus*, *S. epidermidis*, *M. luteus*, *E. coli*, *B. subtilis*, MIC/MBC 600/2500, 300/600, 300/600, 2500/>2500, and 600/600 μg/mL respectively [79] *S. aureus*, MIC: 232 μg/mL, *B. subtilis*, MIC: 464 μg/mL [88].<br><br>*Antifungal:* 2,6-Dimethoxy-*p*-benzoquinone (**20**) [78]. Gallic acid (**21**), *C. albicans* [81] and MIC/MBC 1200/2500 μg/mL [79].<br><br>*Antiviral:* Gallic acid (**21**), EV- 71, IC_{50}: 0.7 μg/mL; HRV-3, 100 μg/mL, 58 %, [83]; HCV, IC_{50}: 15.9 μg/mL [87], HSV-1, IC_{50}: 20 μg/mL [91]; HCV, IC_{50}: 24.3 μg/mL [91]; Influenza A, IC_{50}: 2.6 μg/mL [89]; HCoV, IC_{50}: 71.4 μM [90,93], WSSV [84].
Rhizophora stylosa Griff.				

RUBIACEAE<br>*Gardenia lucida* Roxb.	•	•		*Antifungal:* 5,4′-Dihydroxy-6,7,8,-trimethoxyflavone (**70**), *F. solani*, *A. parasiticus* and *C. tropicalis* [91]<br>*Antiviral:* 5,4′-Dihydroxy-6,7,8,-trimethoxyflavone (**70**), *HIV*, *IC_{50}: 17.4* μM [236].
Guettarda speciosa L.	•			*Antibacterial:* Indole alkaloids, *M. tuberculosis* [230].<br>*Antiviral:* Loganic acid (**67**), IC_{50}: 20 μg/mL, HCV [228]. 4,5-di-*O*-caffeoylquinic acid (**69**) HIV-1, HIV-2, SIM, and HSV, EC_{50}: 0.6, 8, 2, and 0.1 μg/mL, respectively [81,229].
Hydnophytum formicarum Jack				*Antibacterial* Butein (**71**), *M. tuberculosis*, MIC: 12.5 μg/mL [237].<br>*Antiviral:* Butein (**71**), 50 μg/mL, HIV protease by 57.9% [88].
Morinda citrifolia L.	•<br>•	•	•	*Antibacterial:* Damnacanthal (**72)**, *P. aeruginosa,* 10 μg/disc [220]. 1,3-Dihydroxy-5-methoxy-2,6-bismethoxymethyl-9,10-anthraquinone, *S. aureus*, MIC: 24 μM [234]. (*E*)-phytol (**73**), *M. tuberculosis*, MIC: 32 μg/mL [232]. Damnacanthal (**70**), *M. tuberculosis*, MIC: 13 μg/mL [233,93].<br><br>*Antifungal:* Damnacanthal (**72**), *A. ochraceus*, *A. niger*, *C. lipolytica* [91].<br><br>*Antiviral:* 1,3-Dihydroxy-5-methoxy-2,6-bismethoxymethyl-9,10-anthraquinone (**71**), Influenza virus A H1N1 and H3N2, IC_{50}: 10.5 and 11.5 μM, respectively [234]. 1,3-Dihydroxy-5-methoxy-6-methoxymethyl-2-methyl-9,10-anthraquinone, IVA H1N1, IC_{50}: 66.1 μM [234]. Asperuloside (**68**), HSV, IC_{50}: 111.3 μg/mL [234] and EBV [232].
Myrmecodia tuberosa Jack				*Antibacterial:* Iridoids, *S. aureus*, MIC: 100 μg/mL [231].
Psychotria serpens L.	•			
Scyphiphora hydrophyllacea C.F. Gaertn.	•	•		
RUTACEAE<br>*Acronychia pedunculata* (L.) Miq.		•		*Antibacterial:* Acrovestone (**60**) [180].
Atalantia monophylla DC.				

		•		
Limnocitrus littoralis (Miq.) Swingle	•	•		
SALVADORACEAE				
Azima sarmentosa (Bl.) B. & H.	•	•		
Azima tetracantha Lam.	•	•	•	
SAPINDACEAE				
Allophylus cobbe (L.) Raeusch	•	•		
Harpullia arborea (Blanco) Radlk	•	•		
SIMAROUBACEAE				
Quassia indica (Gaertn.) Nooteboom	•	•		
SOLANACEAE				
Solanum viride R.Br.	•	•		
SONNERATIACEAE				
***Sonneratia apetala* Buch.-Ham**.				
***Sonneratia griffithii* Kurtz.**	•			Antibacterial: 3β-Hydroxy-lup-9(11),12-diene, 28-oic acid (**39**) , lupeol (**40**), lupan-3β-ol (**39)**, S. aureus, MIC: 32.2, 15.6, and 33.1 µg/mL, respectively. 3β-Hydroxy-lup-9(11),12-diene, 28-oic acid (**39**) , lupeol (**40**), lupan-3β-ol (**41)**, *S. mutans*, MIC: 35.6, 40.6, and 55.2 µg/mL, respectively [146].
***Sonneratia ovata* Back.**	•			
STERCULIACEAE				
***Heritera littoralis* Aiton**	•			*Antibacterial:* Afzelin (**51**), *S. aureus*, *P. aeruginosa*, *S. typhi*; MIC: 8, 16, and 2 µg/mL, respectively [159]. Astilbin (**54**), *S. sobrinus* MIC/MBC: 225/900 µg/mL [161].<br>*Antifungal:* Afzelin (**51**), *C. parapsilosis*, *C. neoformans*, MIC: 16 and 4 µg/mL, respectively [159]. Taraxerol (**52**), *T. mentagrophytes* and *T. rubrum*, MIC at 12.5µg/mL [55].
.	•			
	•	•		

***Heritiera fomes* Buch. Ham.**				*Antiviral:* Afzelin (**51**), 50 µg/mL, HSV-1 by 72.5% [160]. Friedelin (**53**), HIPV-3, IC_{50}: 14 × 10^{-5} M [162], at 5 µg/mL HCoV by 132.4 % [163].
VERBENACEAE	●			
Premna odorata Blanco				
Premna integrifolia L.				*Antibacterial:* 1-Heneicosyl formate (**86**), *M. tuberculosis*, MIC: 8 µg/mL [252].
VITACEAE				
Cayratia trifolia (L.) Domin				*Antibacterial:* ε-Viniferin (**12**) (200 µL/well from 1 mg/mL solution), *E. coli* and *S.* aureus, 11 and 7 mm, respectively [56]. ε-Viniferin (**12**), MRSA, MIC: 25 µg/mL [57], *S. mutans* and *S. sanguis*, MIC/MBC: 25/50 and 50/50 µg/mL, respectively [54,55]. ε-Viniferin (**12**) *S. aureus* and *P. aeruginosa* , MIC: 512 and 256 µg/mL, respectively [59]. Resveratrol (**14**), *S. mutans* and *S. sanguis*, MIC/MBC: 50/50 and 50/100 µg/mL, respectively, *S. aureus*, MIC: 512 µg/mL [54,55]. *Antifungal:* Resveratrol (**14**), *B. cinerea*, conidia IC_{50}: 90 µg/mL [58]. *Antiviral:* ε -Viniferin (**12**), HCV, EC_{50}: 0.1 µM [54]. IVA, IC_{50}: 88.5 µg/mL [53]. Piceid (**13**), IVA, IC_{50}: 110.7 µg/mL [53].

Bold: true mangrove plants [3]
●: Activity of extract(s) reported in the literature
IZD: Inhibition zone diameter
